# Feline leukaemia virus (FeLV) infection in domestic pet cats in Australia and New Zealand: Guidelines for diagnosis, prevention and management

**DOI:** 10.1111/avj.13470

**Published:** 2025-07-26

**Authors:** ME Westman, SJ Coggins, M van Dorsselaer, JM Norris, RA Squires, M Thompson, R Malik

**Affiliations:** ^1^ Sydney School of Veterinary Science The University of Sydney Sydney New South Wales Australia; ^2^ The Sydney Institute for Infectious Diseases The University of Sydney Sydney New South Wales Australia; ^3^ The Cat Clinic New Town Tasmania Australia; ^4^ College of Public Health, Medical & Vet Sciences James Cook University Townsville Queensland Australia; ^5^ Centre for Veterinary Education The University of Sydney Sydney New South Wales Australia

**Keywords:** antibodies, diagnosis, feline leukaemia virus, guidelines, infection, PCR, review, saliva, treatment, vaccination, veterinary science

## Abstract

Progressive feline leukaemia virus (FeLV) infection dramatically shortens the lives of infected cats, causing acquired immunodeficiency, aplastic anaemia, lymphoma, leukaemia and other myeloproliferative diseases. The potential impact of regressive FeLV infection on the development of disease remains largely unknown, although there is evidence it contributes to lymphoma development. Despite a perception that there has been a general decline in the incidence of progressive FeLV infection in Australia and New Zealand, it remains an important health threat and the risk of infection should not be ignored. Clinicians should therefore have a thorough understanding of the complexities surrounding the diagnosis, management and prevention of this disease. Point‐of‐care (PoC) antigen testing using whole blood is the first step to detect progressive FeLV infection. Clinicians should remember the increased rate of false‐positive results using such kits when the disease being detected is at a low prevalence. We therefore advise that confirmatory FeLV polymerase chain reaction (PCR) testing to detect proviral DNA is essential before a PoC‐positive cat can be confirmed as being FeLV‐infected. Critically, progressively infected cats should not be euthanased because of a positive FeLV diagnosis, as some cats will remain healthy for many years. Regressively infected cats should not be used as blood donors, so blood donor programmes should include FeLV antigen and provirus PCR testing in their standard screening protocols. No cure currently exists for progressive or regressive FeLV infection; therefore, veterinarians should advocate to minimise the exposure of cats to FeLV as a first‐line preventative strategy. The most reliable way to achieve this is for cats to be kept indoors, or with secured outdoor access (e.g., cat enclosures and secure gardens). Cats kept in this manner do not require FeLV vaccination. All animal holding facilities should aim to individually house untested adult cats to limit the spread of FeLV infection. For at‐risk cats that cannot be kept indoors/enclosed, or for cats that live together with known FeLV‐infected cats, vaccination should be undertaken. Two pentavalent vaccines containing inactivated whole‐FeLV are currently available in Australia, whereas no FeLV vaccine is currently available in New Zealand. Given the unavailability of monovalent FeLV vaccines, we endorse the use of a pentavalent vaccine in Australia only in FeLV‐endemic catteries or in situations where there is a demonstrable and substantial risk of FeLV exposure. Manufacturers are encouraged to reintroduce efficacious monovalent FeLV vaccines in Australia and New Zealand. Further research into potential antiretroviral therapy to treat FeLV infections in cats is needed.

Feline leukaemia virus (FeLV) and feline immunodeficiency virus (FIV) are often tested for concurrently, due to the availability of many dual point‐of‐care (PoC) test kits. These retroviruses, however, should be considered by veterinarians to be very distinct pathogens (Table 1). For this reason, we have developed separate guidelines for the diagnosis, prevention and management of FeLV (this document) and FIV.^1^

**Table 1:** Key differences in diagnosis, disease categorisation and prevention between feline leukaemia virus (FeLV) and feline immunodeficiency virus (FIV). Information about FeLV is covered in these guidelines, whereas information about FIV is found in previous guidelines for Australian and New Zealand veterinarians^1^

	FeLV^2^, ^3^, ^6^, ^9^, ^10^, ^11^, ^12^	FIV^13^, ^14^, ^15^, ^16^, ^17^, ^18^
Diagnosis	FeLV diagnosis is predominantly by p27 antigen detection and proviral DNA polymerase chain reaction (PCR) testing.	FIV diagnosis is predominantly by antibody detection and/or proviral DNA PCR testing.
Categories	Progressively infected, regressively infected, focally infected, abortively infected and uninfected.	Infected and uninfected.
Vaccine effectiveness	Two pentavalent vaccines containing inactivated whole‐FeLV are available in Australia which are likely highly effective (~90%; although field efficacy studies are lacking).	One dual subtype (A/D) standalone FIV vaccine is available in Australia and New Zealand. It appears moderately effective in Australia (~56%) and possibly much less so in New Zealand.

FeLV is an enveloped RNA virus in the genus *Gammaretrovirus* of the family *Retroviridae*. Although outcomes of infection vary between cats, the ability of FeLV to cause immune suppression, bone marrow dysfunction, lymphoma and leukaemia has made it an important cause of morbidity and mortality in domestic cats.^2^, ^3^

Various classifications and nomenclature have been used to describe the spectrum of disease observed in cats following FeLV exposure. In the early years after FeLV's discovery in 1964, infected cats were described as “persistently viraemic”, “transiently viraemic” or “regressor cats”.^4^, ^5^ With the advent of molecular testing, the terminology for FeLV‐infected cats changed to “progressively infected”, “regressively infected” (with or without an antecedent transient viraemia), “focally infected” and “abortively infected”.^6^

An additional classification scheme intended to augment this approach has been adopted by one diagnostic laboratory (IDEXX), based on a prospective study of FeLV‐infected cats.^i^ Using diagnostic test results, a cat's infection status is classified as “high positive” or “low positive”. “High positives” are likely progressive infections, whereas “low positives” are likely regressive and focal infections.^7^ This scheme aims to establish a more practical approach to the staging and monitoring of FeLV infections, using qualitative p27 antigen and quantitative proviral DNA test results.^8^ An inverse correlation to survival time has been demonstrated, such that higher proviral loads (i.e. progressive infections) are correlated with poorer outcomes.^7^ This approach has been adopted by IDEXX Australia but not yet by IDEXX New Zealand (see Section 3 – “Diagnosis of FeLV infection”).

## Pathogenesis and categories of FeLV infection

These guidelines will adopt the “progressive”, “regressive”, “focal” and “abortive” nomenclature, as it is the most widely accepted terminology.^6^, ^19^

Clinicians in Australia and New Zealand should be aware of these four categories of FeLV infection, but will likely be concerned largely with progressively infected cats. Rarely, they may diagnose a regressively infected cat with serial antigen testing (see Section 3 – “Diagnosis of FeLV infection”).

It is essential that all blood donor programmes test to identify and remove any progressively or regressively infected cats (see Sections 3 and 4 – “Prevention of FeLV infection”).

Progressively infected cats can readily transmit the virus to FeLV‐naïve cats, usually via transfer of infected saliva. Close, “friendly” contact between cats (e.g., suckling, mutual grooming, nose‐to‐nose contact and sharing of food/water bowls/toys) leads to much of the horizontal transmission. Infected queens regularly infect their kittens in utero or after parturition via grooming or infected milk.^19^ Given the high concentration of FeLV in saliva, deep bites acquired from fighting, for example in free‐roaming cats, may also facilitate viral transmission^19^, ^20^, ^21^ although the epidemiology observed in Australia does not favour this mode of transmission. Blood transfusions are another potential pathway for FeLV transmission,^22^ as is the use of dental and surgical instruments that have not been appropriately sterilised between procedures.

FeLV exposure and infection can lead to a spectrum of possible outcomes. The final position on the FeLV “consequence spectrum” is a balance between:

1. the cat's humoral and/or cell‐mediated immune response (affected by breed, age of the cat when first exposed, concurrent drug therapy, any concurrent disease(s) or other stressor(s) affecting the immune status of the cat);
2. the magnitude of the viral challenge; and
3. possibly the specific infecting virus strain.

**Figure 1:**
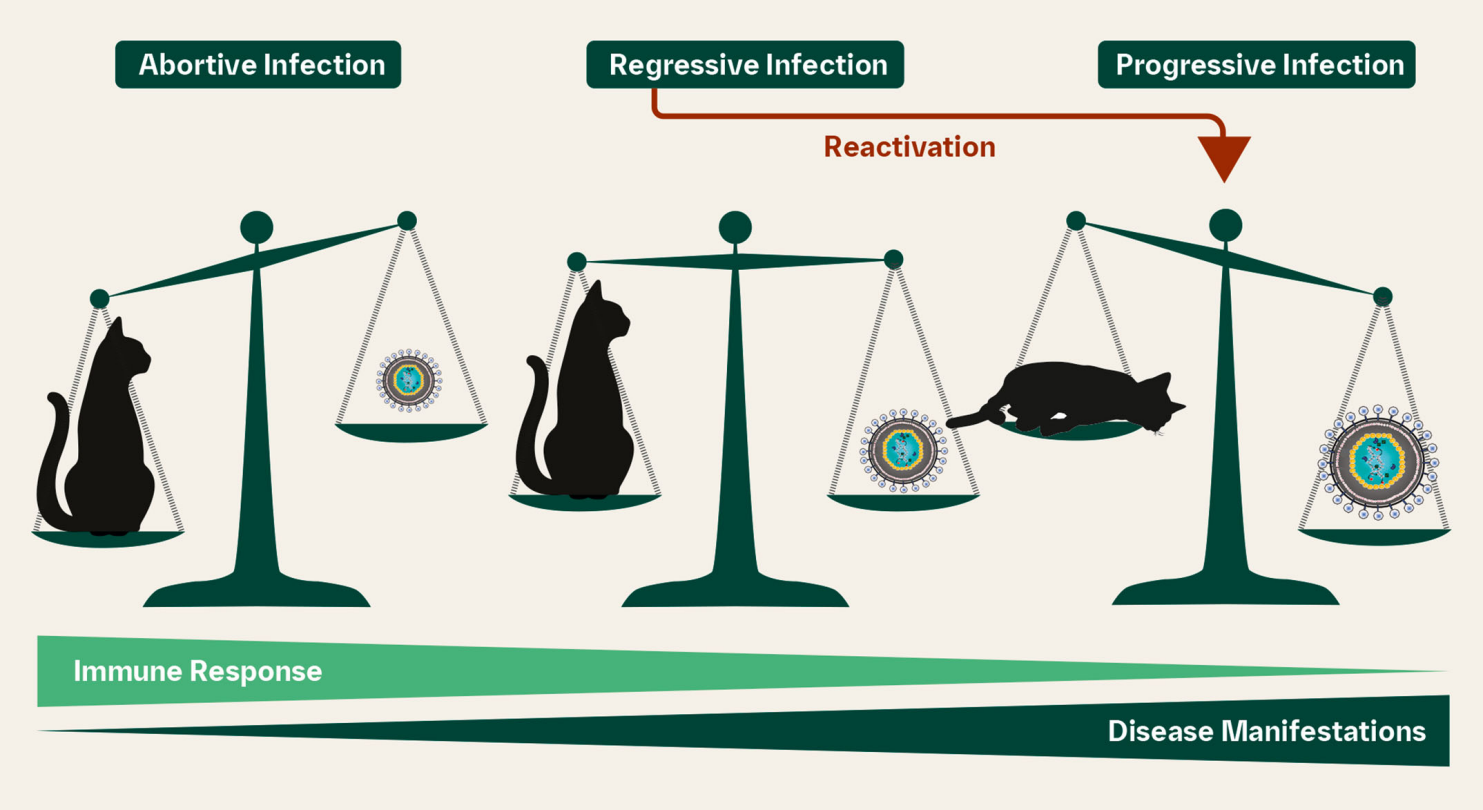
FeLV infection causes a spectrum of disease, with the outcome a balance between the cat's immune response and the virus. Cats with abortive and regressive infections should be assumed to have the same status as an uninfected cat and are likely to have a normal lifespan. Progressively infected cats have a higher viral load and poorer prognosis, with reported mortality rates of up to 90% within 3 years of acquiring infection. Adapted from Hofmann‐Lehmann and Hartmann (2020).^6^

Cats with progressive infections have a poor immune response that allows FeLV to continuously replicate and eventually overcome host defence mechanisms to cause disease; cats with regressive infections have a partially effective immune response; and cats with abortive infections have a strong immune response that enables them to “win the battle” over FeLV (Figure 1).^6^

### Progressive infections

Progressively infected cats have a poor immune responsiveness to FeLV, resulting in permanent (lifelong) and persistent viraemia. In these cats, after exposure to FeLV, local replication first occurs in the oropharyngeal tissue and progresses via infection of monocytes and lymphocytes to a primary viraemia usually lasting 2–16 weeks. After this, a secondary (and lifelong) viraemia occurs, with infection of leukocyte progenitor cells in the bone marrow (granulocytes, mononuclear leukocytes and platelet precursors).^19^, ^23^ Additionally, FeLV replication also occurs in a range of other host tissues including lymph nodes, salivary glands, spleen, intestines, kidneys and bladder.^6^, ^24^ Consequently, progressively infected cats persistently shed viable virus in secretions and excreta, especially saliva, but also faeces, urine and milk.^6^, ^25^, ^26^, ^27^, ^28^

**Figure 2:**
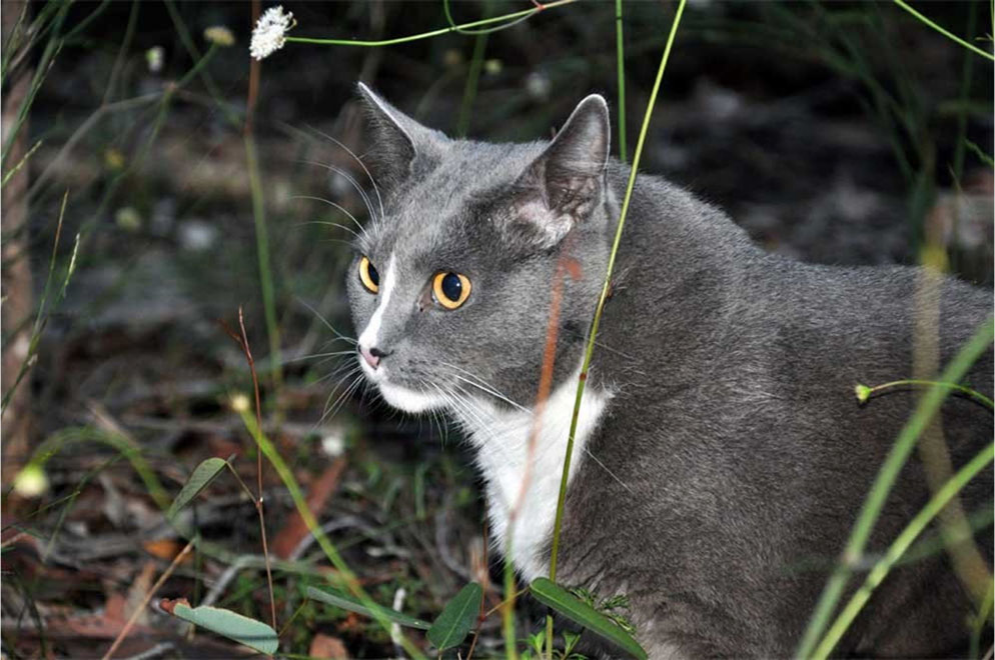
“Pinky” was diagnosed with progressive FeLV infection at 11 years of age. Despite the age‐related resistance that exists with FeLV challenge (younger cats are more susceptible than older cats), adult cats can and do become infected with FeLV. “Pinky” was later euthanased due to progression of chronic kidney disease. Image kindly provided by Lynda du Cross.

Although “age resistance” remains important in FeLV pathogenesis and epidemiology, adult cats can and do become progressively infected with FeLV (Figure 2).^19^ For example, in a study of 18,038 cats sampled at 345 American veterinary clinics in 2004, 2.6% were found to be FeLV antigenaemic, of which adult cats (>6 months old) were 2.5 times more likely to be progressively FeLV‐infected than juvenile cats (<6 months old).^29^ In a recent FeLV treatment case series in Australia, there was a bimodal age distribution: 10 of 18 cats were less than 4 years of age, and 8 of 18 cats were over 6 years of age.^30^

Progressively infected cats have a poor prognosis, with reported mortality rates of up to 90% within 3 years of acquiring infection.^3^, ^12^, ^24^, ^31^ Mortality is predominantly due to aplastic anaemia, lymphoma (most commonly mediastinal lymphoma), leukaemia or other myeloproliferative diseases.^3^, ^12^, ^24^, ^31^, ^32^, ^33^, ^34^, ^35^, ^36^, ^37^ A trend for decreasing incidence of FeLV‐associated lymphoma in many parts of the world has changed the typical feline lymphoma presentation from FeLV‐infected younger cats with mediastinal lymphoma to FeLV‐uninfected older cats with intestinal or extra‐nodal lymphoma (solitary, nonlymphoid organ involvement, e.g., nasopharynx, central nervous system, kidneys and skin).^38^, ^39^

In the recent FeLV treatment case series in Australia, a median survival time of 634 days (1.7 years) from treatment of cats with progressive infections and a poorer survival in young FeLV‐infected cats compared with older FeLV‐infected cats were reported. For every 1‐year increase in age at diagnosis, survival time increased by 88 days. Thus, younger cats fared considerably worse than older cats. This finding was likely associated with a higher “viral load” in younger cats than older cats: for every 10‐fold increase in initial viral load, survival time decreased by 1.8 years.^30^

### Regressive infections

Regressively infected cats have a partially effective immune response against FeLV that clears a primary viraemia, typically within 4 months beyond initial infection, to prevent a secondary viraemia. The immune response is insufficient to prevent lifelong infection, however, due to a DNA copy of the RNA virus (called provirus) becoming integrated into the cat's own cellular DNA.^3^ Provirus is found in the blood, usually in lymphocytes and occasionally monocytes, with a range of other host tissues also possibly infected including, uncommonly, the bone marrow.^6^, ^24^ Once viraemia has cleared, these cats are not contagious to FeLV‐naïve cats and therefore pose no risk in group‐housing situations. Some cats actually bypass the viraemic stage, meaning they are never detectably antigenaemic (p27‐positive; see Section 3 – “Diagnosis of FeLV infection”).^40^, ^41^

The contribution of regressive infections to the development of FeLV‐related disease remains to be investigated. An association between regressive infections and lymphoma has been observed in some countries, including Australia and Canada, and over a 20‐year timespan.^42^, ^43^, ^44^, ^45^, ^46^ Other reviews have suggested that cats with regressive infections have a similar life‐expectancy to FeLV‐uninfected cats and that FeLV‐associated disease is unlikely to develop.^2^, ^6^, ^12^ One study followed three regressively infected cats for more than 12 years after experimental inoculation and did not report any FeLV‐associated disease.^24^ Further long‐term studies are required to verify which prognosis is most accurate. FeLV proviral DNA PCR testing after a negative antigen test result to detect regressive infections in cats with lymphoma is not routinely performed in Australia or New Zealand, nor anywhere else in the world. Therefore, clinical records cannot easily be interrogated retrospectively to look for a disease association (see Section 3 – “Diagnosis of FeLV infection”). To prove causation, elegant cancer genomic studies or a prospective case–control study of regressively infected FeLV cats compared with age‐matched FeLV‐uninfected controls, are required.

Regressively infected cats are capable of transmitting FeLV to other cats via blood transfusions and therefore should never be recruited as blood donors.^22^, ^47^ Regressive infections represent another group of FeLV‐infected cats that should be considered when performing risk analyses for preventative healthcare strategies and vaccine protocol choices.

### Focal (localised, atypical) infections

Cats with focal infections, as the name suggests, have a robust immune response able to restrict FeLV replication to particular tissues such as spleen, lymph nodes, small intestine, urinary tract or mammary glands.^6^, ^28^, ^48^ Initially thought to occur rarely in the field,^49^ recent Australian and European studies have challenged this notion.^50^, ^51^ Literature describing focal infections is limited, and some reported cases were not followed longitudinally; thus, clinical signs associated with focal infection at this stage are speculative and difficult to predict.

### Abortive infections

Abortively infected cats mount an effective immune response that, after localised replication in the oropharyngeal lymph nodes, results in complete elimination of FeLV. They are not infectious to other cats. FeLV‐related disease does not develop, and these cats have the same lifespan as FeLV‐unexposed cats.^6^, ^19^ Like regressively infected cats, this group of exposed cats is again important when performing risk analyses for preventative healthcare strategies and considering vaccine choices. A complete FeLV risk assessment cannot be achieved without identifying all outcomes of FeLV exposure in a population of cats. Clinicians in Australia and New Zealand are currently unable to identify abortive infections due to limited FeLV testing options (see Section 3 – “Diagnosis of FeLV infection”).

## Prevalence of FeLV infection

In both Australia and New Zealand, FeLV infection remains an uncommon, yet highly preventable, cause of morbidity and mortality in pet cats.

We suspect, however, that FeLV exposure and infection are more common than many clinicians realise.

**Figure 3:**
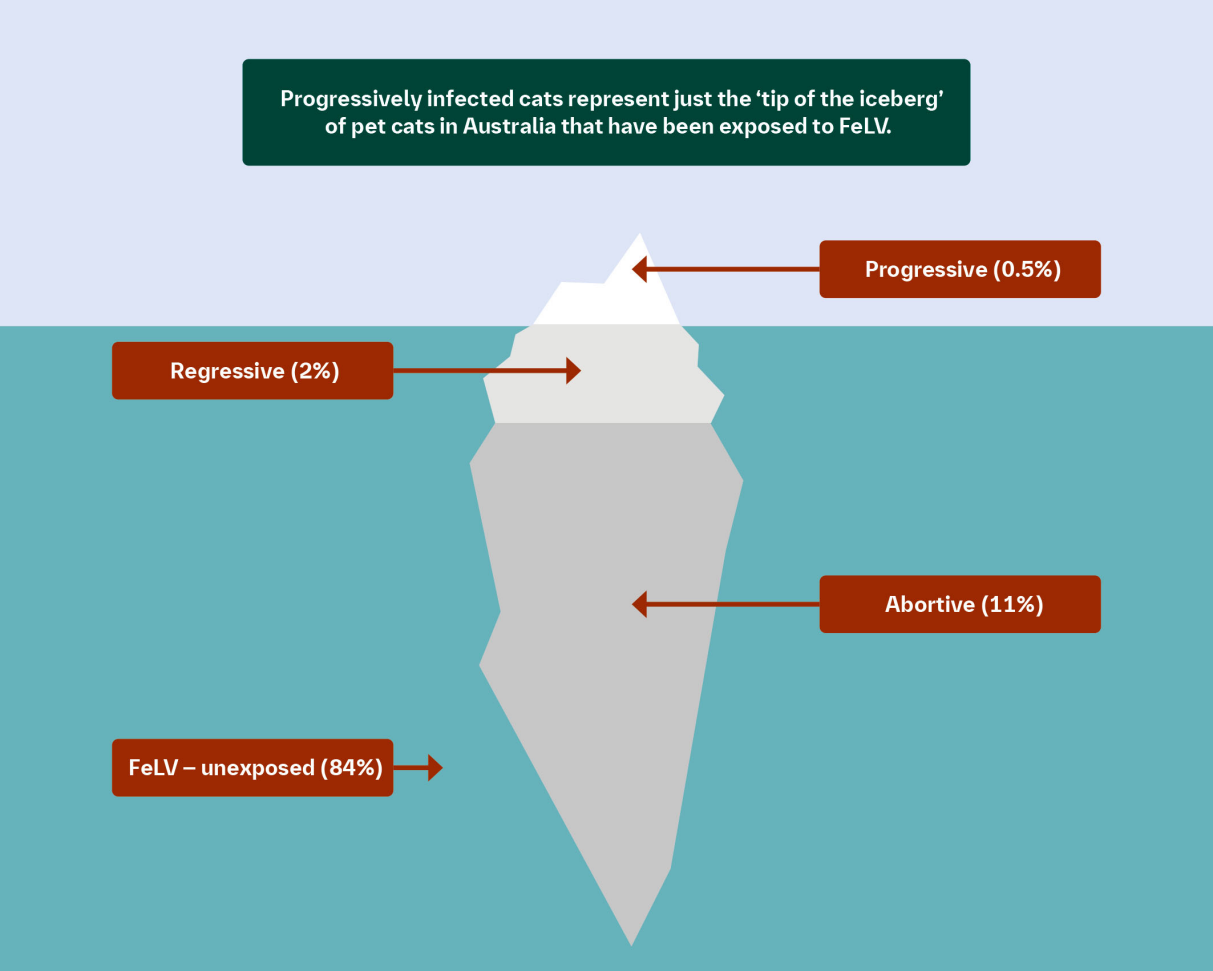
FeLV infection status of healthy, client‐owned cats with outdoor access predominantly from Eastern Australia. Approximately 2.5% of cats were unclassifiable as FeLV‐unexposed or abortive due to insufficient samples being available for further antibody testing.^51^ Not to scale.

In Australia, progressively infected cats may represent only the “tip of the iceberg” of pet cats that have been exposed to FeLV. Regressive and abortive infections also need to be considered to understand fully the risks posed by FeLV in a cat population and the overall viral dynamics (Figure 3).

A perception that there has been a general decline in the incidence of progressive FeLV infection in Australia and New Zealand exists amongst many older clinicians. This is despite many differences in methodologies between studies in both countries, including recruitment criteria (e.g., age, healthy vs. sick cats, risk factors such as lymphoma), FeLV tests performed (e.g., immunofluorescent antibody, ELISA, immunochromatography and PCR testing) and the study recruitment area. We, however, concur with this view.^52^, ^53^

Clinicians in Australia and New Zealand screen for FeLV infection using rapid PoC test kits, meaning they usually only detect cats with a persistent viraemia (antigenaemia) and therefore progressive infection (see Section 3 – “Diagnosis of FeLV infection”). This section thus focuses on recent surveys investigating the prevalence of progressive infection in these two countries. Additional historical context, and further information pertaining to the prevalence of regressive and abortive infections in Australia, can be found in the Supplementary Materials (S1). Investigations into the prevalence of regressive and abortive infections in New Zealand are lacking and needed.

### Prevalence of FeLV infection in Australia

In a cohort of healthy pet cats in Australia aged 2 years of age or older with some level of outdoor access, sampled during 2013–2015, progressive infections were found to have a prevalence of 0.5% (2/440). Cats included in the study lived in or near the cities of Adelaide, Melbourne, Canberra, Sydney and Brisbane. A small cluster of FeLV exposure in south‐western Sydney, encompassing an area between Liverpool and Campbelltown, was identified. This study demonstrated that progressive FeLV infections still occur, albeit uncommonly, in pet cats in Australia.

In the same survey, a prevalence of 2% (9/440) regressive infections and 11% (47/440) abortive infections were identified. This meant regressive infections were 4.5 times more common than progressive infections, and abortive infections were 23.5 times more common than progressive infections in the same cohort. This result indicated that FeLV exposure might be more prevalent in Australia than previously realised but under most circumstances, infections after exposure were abortive or regressive (Figure 3).^51^ The most likely explanation for this finding would be frequent exposures, but each with a very small viral inoculum, or exposure when cats were relatively resistant via age‐related resistance.^54^

Australia is a large country and the prevalence of FeLV infection has been shown to vary between regions. It has been suggested that FeLV infection is more common in Western Australia (WA).^55^, ^56^ The situation regarding the prevalence of progressive FeLV infection in pet cats in much of Australia, however, remains unknown. Opportunistic PoC testing at a clinic in Wagga Wagga, NSW (a relatively large regional city) with Anigen Rapid® FeLV kits produced 4.5% (2/44) FeLV antigen‐positive results, but confirmatory FeLV PCR testing for proviral DNA was not performed (unpublished data).

Two substantial FeLV outbreaks in small Sydney‐based rescue facilities have been documented over the past decade. Both rescue groups did not routinely test new arrivals for FeLV and practiced some level of group‐housing. This created a “perfect storm” for FeLV transmission, with lots of vulnerable, immunologically naïve kittens exposed to progressively infected cats. After the identification of index FeLV cases, testing was undertaken to help quickly identify all FeLV‐infected cats. In total, progressive infections were discovered in 21% (19/89) of cats, regressive infections in 25% (22/89) of cats and abortive infections in 9% (8/89) of cats.^51^ Some kittens were rehomed before the outbreaks were detected and brought under control, meaning some unknowingly FeLV‐infected kittens ended up in private homes. This was a reminder that veterinarians should always include in their history‐taking a question about the origin of the animal and whether the retroviral status of the animal is known.^19^

The prevalence of progressive FeLV infection in cats presenting with lymphoma in Australia has also declined over time. Nowadays, progressive FeLV infections are rarely diagnosed in cats with lymphoma in Australia. In a recent study of abdominal lymphoma in Australian cats from 2017 to 2021, 0/34 (0%) cats tested positive for FeLV antigen.^57^ A retrospective study of lymphoma in Australian cats from 2000 to 2022 seen at two referral centres located in Melbourne and Sydney found 4.6% (13/284) cats tested positive for FeLV^58^; the majority of FeLV testing performed was SNAP® PoC testing, with very few confirmatory PCR tests performed, so it is unclear how many of these were false‐positive results (Peter Bennett, pers. comm.).

More FeLV testing needs to be done and reported in other cities and regional centres in Australia.

### Prevalence of FeLV infection in New Zealand

Although published data are lacking, clinicians who practiced in New Zealand in the 1970s and 1980s recollect seeing far more FeLV‐related disease among New Zealand pet cats than more recently. In 2015, a cross‐sectional survey of 112 veterinary practices found that 2.6% (56/2125) of PoC test results were positive for FeLV.^59^ At a first‐opinion practice in Waimate, the overall FeLV positivity rate with SNAP® testing between 2010 and 2016 was 7% (41/572); but of these 41 FeLV‐positive cats, only two had confirmatory PCR testing performed, with 1/2 cats (50%) testing PCR‐positive.^59^ Cats entering the New Zealand SPCA shelter in Auckland in 2014 had a FeLV positivity rate with SNAP® testing of 1% (4/388); 2/4 (50%) of the FeLV‐positive cats were PCR‐positive on confirmatory testing.^60^

Anecdotally, pockets of FeLV infection have been reported in Taranaki, Queenstown and Waikato (Natalie Lloyd, Zoetis Animal Health, pers. comm.). A feline medicine specialist based in Wellington reports diagnosing occasional progressive FeLV infections in kittens and cats from rescue cases from the Wairarapa and Kapiti Coast and recently provided advice on a progressive FeLV infection in a Burmese kitten from a “backyard breeder” (Pru Galloway, pers. comm.).

Studies in New Zealand reporting the FeLV status of cats presenting with lymphoma and investigating the presence of FeLV proviral DNA in the tumours have not yet been performed.

## Diagnosis of FeLV infection

Australian and New Zealand veterinarians have access to a range of PoC test kits to detect FeLV antigen in whole blood, plasma or serum.

**Figure 4:**
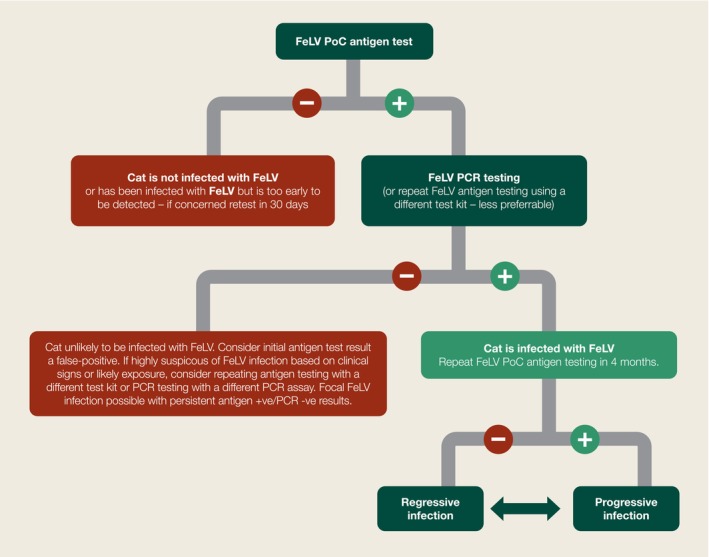
Suggested decision‐making algorithm for diagnosis of FeLV infection in Australia and New Zealand. Cats with FeLV infection may have alternating test results that fluctuate between regressive and progressive, especially early in the course of disease and with a changing immune status (e.g., due to age or comorbidities). With repeated testing over time, a definitive regressive or progressive outcome usually eventuates.^6^, ^19^ PoC, point‐of‐care.

Proviral DNA PCR testing to rule out PoC false‐positives is essential (Figure 4). One confirmatory PCR test per animal or per household might be sufficient.

Since regressive infections can also test antigen‐positive early in the course of infection, repeat PoC testing 4 months later should always be performed to differentiate progressive infections (cats are persistently antigen‐positive) from regressive infections (cats become antigen‐negative).

It is useful to know the FeLV (and FIV) status of any new kitten or cat upon entry to a shelter or new household.

In particular, FeLV testing before admission to group‐housing situations (catteries, rescue facilities, shelters and multicat households) should be mandatory to prevent avoidable disease outbreaks.

Neither FeLV antigen testing nor PCR testing is affected by maternal antibodies in kittens or by FeLV vaccination. Therefore, unlike FIV testing, FeLV testing can be performed in kittens of any age, and in cats irrespective of FeLV vaccination history.^6^, ^19^

All blood donor cats should be screened for FeLV infection before donation using both PoC and PCR testing.

Progressive FeLV infection is typically simple to diagnose. Occasionally, however, diagnosis is not straightforward. FeLV‐infected cats may have alternating test results that fluctuate between regressive and progressive infection status, especially early in the course of disease, and with changing immune status (e.g., due to advancing age or developing comorbidities). With repeated testing over time, a definitive outcome—either regressive or progressive infection—usually eventuates.^6^, ^19^

To diagnose and categorise FeLV‐infected cats, Australian and New Zealand veterinarians currently have access to two types of testing:

1. Antigen testing to detect soluble FeLV capsid protein (p27) in blood using rapid PoC tests and
2. PCR testing to detect FeLV proviral DNA in blood or bone marrow (i.e., virus inserted into the host genome).

Figure 4 summarises a practical approach to FeLV testing and FeLV infection classification for Australian and New Zealand veterinarians using PoC and PCR testing. Similar diagnostic approaches have been suggested in the United Kingdom and the United States.^19^, ^61^ Table S1 provides prices for the different FeLV PoC and PCR tests available in Australia.

Information on other FeLV tests available elsewhere in the world including immunofluorescent antibody (IFA) testing, neutralising antibody (NAb) testing, virus isolation and RT‐PCR testing to detect FeLV RNA in saliva is also included in the Supplementary Materials (S2). Table S2 summarises how results from PoC testing, proviral DNA PCR testing and NAb testing can be used to assign progressive, regressive, focal and abortive infections.

### PoC p27 antigen testing to diagnose FeLV infection

To date, the performance of three commercially available FeLV PoC test kits has been independently validated under Australian conditions, using PCR testing for proviral DNA as the reference test (Table 2). The same three FeLV test kits are also available in New Zealand.

**Table 2:** Results from a population of Australian cats comprising 45 progressively FeLV‐infected cats and 491 FeLV‐uninfected cats using blood as the diagnostic specimen. Taken from^62^

	SNAP® FeLV	Witness® FeLV	Anigen rapid® FeLV
	(IDEXX, Westbrook, ME, USA)	(Zoetis Animal Health, San Diego, CA, USA)	(BioNote, Gyeonggi‐do, Korea)
Specificity	94%	98%	98%
Sensitivity	100%	91%	91%

The difference in specificity between Anigen Rapid® FeLV (98%), Witness® FeLV (98%) and SNAP® FeLV (94%), meant that testing with SNAP® produced significantly more false‐positive results than testing with Anigen Rapid® or Witness®. There was no statistically significant difference in sensitivity between test kits, meaning no difference in the rate of false‐negative test results was obtained.^63^

Veterinarians should remember the effect disease prevalence has on the positive predictive value of testing (PPV; proportion of individuals with a positive test result that are truly infected). Since the prevalence of progressive infection in the healthy population in Australia and New Zealand is much lower than in other parts of the world (e.g., 0.5% in Australia vs. 2.3% in Europe), the PPV of FeLV PoC testing in cats is lower in Australia than it is under European conditions.^51^, ^64^ The Australian study reported the PPV of SNAP®, Anigen Rapid® and Witness® PoC test kits to be between 62 and 80%, using PCR testing as the reference test. In other words, up to 38% of positive FeLV PoC test results were false‐positive results. Although PPV will increase as disease prevalence increases, for example only testing cats with clinical signs of FeLV‐related disease, in the same study multiple false‐positive antigen test results were obtained in cats displaying clinical signs consistent with FeLV disease.^63^ Studies in New Zealand have reported a false‐positive rate of 50% with FeLV PoC testing alone.^59^, ^60^

Similarly, in the United States, a 19% false‐positive rate (i.e., approximately one in five cats), based on a single FeLV PoC test result, was reported in 801 cats being rehomed from an adoption programme in Texas.^65^ In a case series involving 18 cats with immune‐mediated haemolytic anaemia enrolled in Italy, Spain and the United Kingdom, discordant results between p27 antigen testing with SNAP® and proviral DNA PCR testing of peripheral blood were reported. All 18 cats were considered to have had false‐positive p27 results, with negative SNAP® results in nine cats subjected to follow‐up testing.^66^ A similar p27 antigen false‐positive phenomenon was also reported in Canada in cats with haematological disease.^67^

Negative predictive values (NPV; proportion of individuals with a negative test result that are truly uninfected) are generally high in populations with low disease prevalence; the NPV of all three FeLV kits evaluated in Australia was comparably high (94%). Thus, negative FeLV results with any of these test kits are likely true‐negatives.^63^

Early in the course of FeLV infection, proviral loads for progressive and regressive infections are similar. Therefore, p27 antigen testing should be repeated 4 months later with a FeLV PoC kit for final determination of the category of FeLV infection.^6^, ^40^ During this time, FeLV‐positive cats must be separated from FeLV‐negative unvaccinated cats to prevent transmission until regressive status is assigned (and therefore the threat of transmission has ceased) or FeLV vaccination of in‐contact cats has occurred. If this follow‐up testing is not performed, some regressively infected cats will be misdiagnosed as being progressively infected.

Antigen (p27) testing performed in‐house at IDEXX Australia and IDEXX New Zealand uses a SNAP® FeLV PoC kit (i.e., the same test available to clinicians).

**Figure 5:**
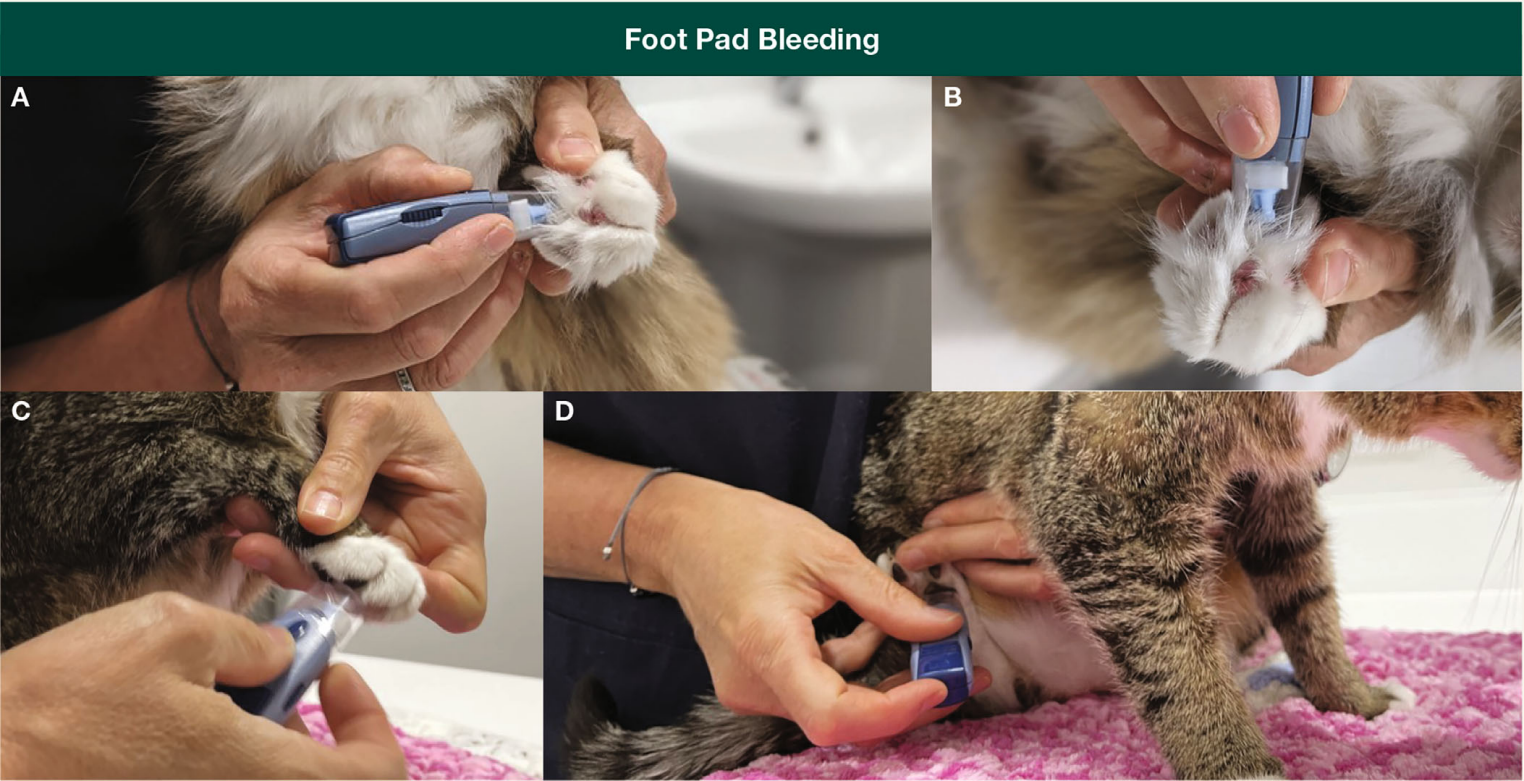
Suggested procedure for foot pad bleeding and FeLV point‐of‐care (PoC) testing. The patient is gently restrained in whatever way is most comfortable. Both metacarpal (A–C) and metatarsal foot pads (D) can be used, depending on which is less stressful for the cat. A lancet device is used to obtain a capillary blood sample by puncturing the skin of the foot pad. First, desensitise the pad by applying a topical local anaesthetic (lignocaine) cream or ointment, ideally at least 60 min prior. Most lancet devices allow for selection of the depth of penetration, and in the case of foot pad collection, a slightly deeper penetration is usually required. After lancing, the foot pad is squeezed gently to produce a drop of blood that is placed directly onto the PoC test strip. The test is then performed as per the manufacturer's instructions, using the recommended amount of buffer. Note that direct application of blood onto the test strip may not be consistent with some manufacturers' instructions that advise mixing with test buffer in a separate tube first. Distracting the cat during sample collection with patting or treats is usually all that is required, with most cats barely aware of the sample being taken. Images provided by Dr Moira van Dorsselaer.

**Figure 6:**
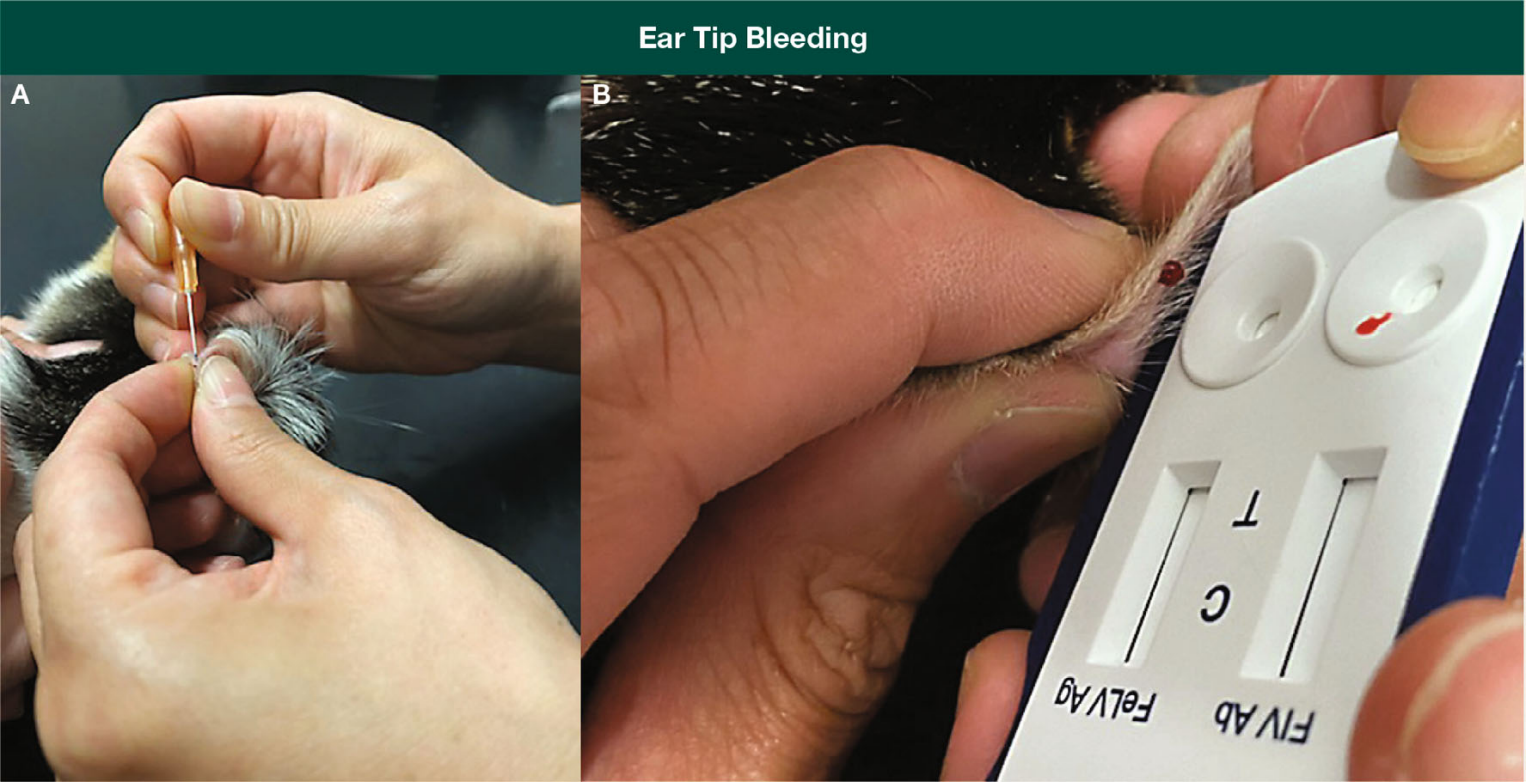
Suggested procedure for ear tip bleeding and FeLV point‐of‐care (PoC) testing, adapted from ear prick sampling for blood glucose testing. Ideally, a vein on the dorsal (outside) surface of the pinna is used for sampling; alternatively, if a vein cannot be seen (e.g., dark‐coloured cats), the medial (inside) surface of the pinna can be used (A). First desensitise the ear by applying a topical local anaesthetic (lignocaine) cream or ointment, ideally at least 60 min prior. The pinna should be massaged before sampling to encourage blood flow to the tip of the pinna. The skin is pricked with a 25 g needle, and a drop of blood squeezed directly onto the test kit strip (B). The test is then performed as per the manufacturer's instructions, using the recommended amount of buffer. Note that direct application of blood onto the test strip may not be consistent with some manufacturer instructions that advise mixing with test buffer in a separate tube first. Gentle compression of the pinna puncture site afterwards will stop the bleeding. Fractious cats can be gently restrained using a towel wrap while sampling is performed. Images kindly provided by Dr Jeffrey So.

“Welfare‐friendly” blood sampling via ear tip or foot pad bleeding is a lower stress option for FeLV PoC testing of some “healthy” cats, for example, before FeLV vaccination (Figures 5 and 6).

### Proviral DNA PCR testing to diagnose FeLV infection

**Figure 7:**
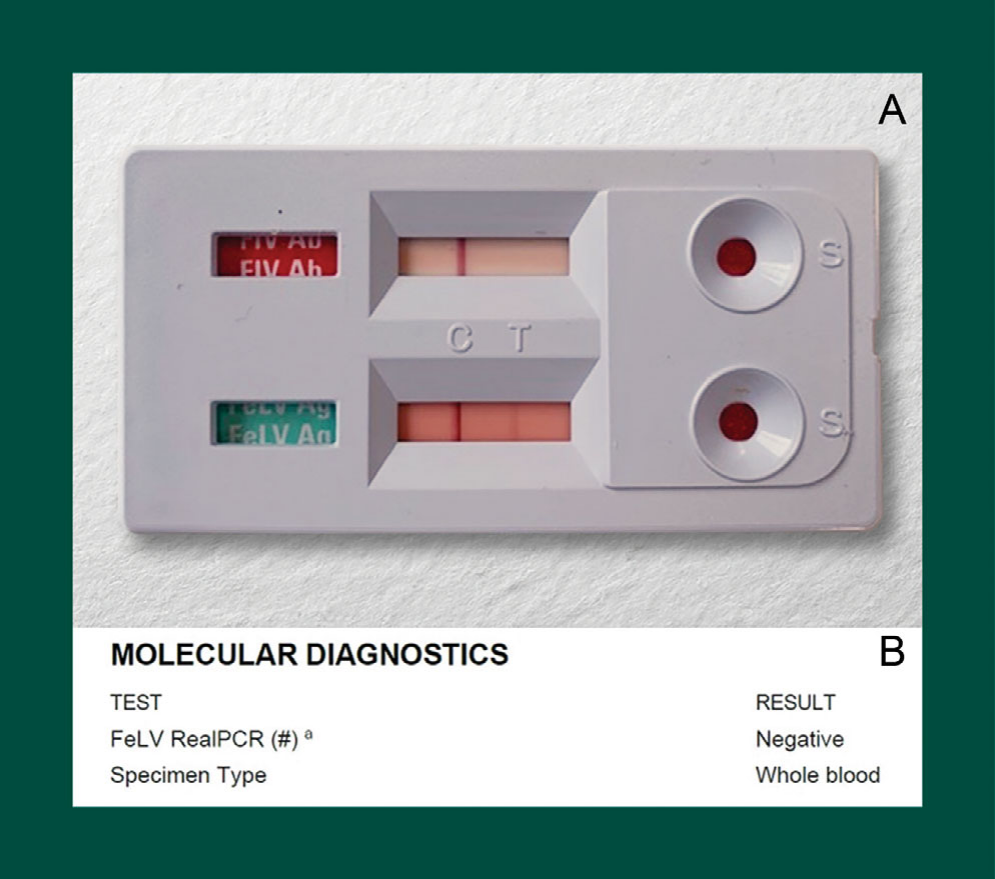
A real‐life example of a false‐positive p27 antigen point‐of‐care (PoC) result from Hobart, Australia. (A) The top strip of this test kit is for FIV antibody testing and the bottom strip is for FeLV antigen testing. The faint positive FeLV PoC result obtained (one dark “control” band and one lighter “test” band on the bottom strip) was assumed to be a false‐positive FeLV result after a (B) negative FeLV PCR result for proviral DNA (FeLV RealPCR™). The cat, a 2‐year‐old male domestic short‐haired cat, was thus considered to be FeLV‐uninfected.

The risk of obtaining a false‐positive FeLV PoC antigen result, and the consequences of a positive FeLV PoC result, mean that initial diagnosis of FeLV infection should never rely solely on a single PoC test result. Confirmatory testing should always be pursued, even in cats presenting with clinical signs consistent with FeLV infection. Otherwise, some FeLV‐uninfected cats will be misdiagnosed as being FeLV‐infected (Figures 4 and 7).

Given there is no reference test for FeLV diagnosis, we advise using FeLV proviral DNA PCR testing of blood as a quasireference test and advise PCR testing over repeat PoC testing for confirmatory FeLV testing. This contrasts with our recommendations for the diagnosis of FIV infection, where we advise using results from two different FIV PoC (antibody) test kits rather than a single PoC test kit and PCR testing.^1^

FeLV PCR testing is available from several external laboratories in Australia and New Zealand and all tests target FeLV proviral DNA (not viral RNA).^ii^ Currently, none of the assays offered by these laboratories has been independently validated.

With IDEXX FeLV Quant RealPCR™ testing in Australia, the “cycle threshold” (C_T_) value is used to quantitate the amount of proviral DNA present in the sample (copies/mL) based on a standard curve, and the result reported as a concentration. This result is then used to classify the cat's result as consistent with progressive infection (≥1.0 × 10^6^ provirus copies/mL; i.e. “high positive”), or consistent with regressive or focal infection (<1.0 × 10^6^ provirus copies/mL; i.e. “low positive”). The breakpoint used by IDEXX for FeLV Quant RealPCR™ test results (1.0 × 10^6^ provirus copies/mL) has gone through an internal validation process^68^ and is slightly higher than the breakpoint used for a survival study in naturally FeLV‐infected cats (4.0 × 10^5^ provirus copies/mL).^7^ IDEXX acknowledges that some cats can fluctuate between progressive and regressive infection, and that testing at a single point‐in‐time may be insufficient to determine the long‐term outcome of FeLV infection in a cat.^8^ This is consistent with all major retroviral testing guidelines (and our recommendations) which advise that repeated antigen testing is required to clearly identify the course of infection.^6^, ^12^, ^19^ IDEXX reports that progressive infections (“high positives”) are more likely to be infectious to other cats, are at increased risk of FeLV‐associated diseases, and prognosis is variable depending on the presence of any concurrent disease(s) or other stressor(s) affecting the immune status of the cat. Cats with regressive or focal infections (“low positives”) have a higher probability of long‐term survival.^8^

FeLV Quant RealPCR™ testing (i.e., reporting a quantitative proviral DNA load present in a sample) is not currently provided by IDEXX New Zealand. Instead, a qualitative PCR‐positive or PCR‐negative FeLV result is reported in New Zealand (FeLV RealPCR™ testing).

Rarely, FeLV PCR testing of bone marrow (in addition to blood testing) may be required for a final diagnosis.^69^, ^70^ Seeking specialist advice in such cases might be warranted.

### FeLV testing using saliva and conjunctival swabs

Saliva testing to detect FeLV p27 antigen is appealing since saliva contains on average five times more infectious FeLV per mL than plasma.^27^ We do not currently recommend PoC testing of saliva for the diagnosis of FeLV infection due to the devastating consequences of missing progressively infected cats, particularly in shelters and group‐housing situations.^6^, ^12^, ^19^, ^62^ Three commercially available FeLV PoC antigen test kits (Anigen Rapid®, Witness® and SNAP®) were tested using saliva instead of blood in Australia and found to have excellent specificity (100%), but less than ideal sensitivity (82%).^63^

Recently, PCR testing to detect FeLV proviral DNA in conjunctival swabs has been demonstrated to be highly accurate in two studies overseas. In both studies, all progressive infections were correctly identified with conjunctival PCR testing, and most regressive infections also tested PCR‐positive.^71^, ^72^ This may represent another welfare‐friendly option for testing, particularly in healthy cat populations when blood collection might be impractical. Validation of this testing method in Australia and New Zealand would be helpful.

### FeLV testing recommendations

Table 3 summarises general FeLV testing recommendations including before FeLV vaccination in Australia. If FeLV testing is not performed before initial FeLV vaccination, and FeLV infection is diagnosed later in life, it will be impossible to determine if the cat was FeLV‐infected before vaccination or if a “vaccine failure” occurred. FeLV vaccination of FeLV‐infected cats has no demonstrated therapeutic benefit.^73^ This is not currently a consideration in New Zealand since no FeLV vaccine is commercially available (see Section 4 – “Prevention of FeLV infection”).

**Table 3:** When and how to diagnose progressive and regressive FeLV infection in Australia and New Zealand

FeLV testing indication	Time of testing	Initial testing	Confirmatory testing
Unwell cat with possible FeLV‐associated disease	Concurrently with other blood tests (e.g. haematology and biochemistry) and before any therapy being commenced	PoC testing using blood to detect p27 antigen	PCR testing using blood to detect proviral DNA
Any new kitten or cat	At time of initial health check, before introduction to a new household (kittens can be tested at any age)		
Before FeLV vaccination (Australia only)	At time of health check and before first FeLV vaccine administration (regardless of age), and before lapsed annual FeLV revaccination		
Cat fight abscess	4 weeks later		

Blood donation (only enrol cats with no possibility of cat fights in the previous 4 weeks)	During screening, before collecting transfusion blood (will also need FIV testing)	Both PoC and PCR testing using blood should always be performed (since regressively infected cats should not be used as blood donors)	

## Prevention of FeLV infection

Vigorous preventative efforts should continue wherever FeLV infection and its associated adverse consequences remain prevalent.

Minimising the exposure of cats to FeLV by keeping them 100% indoors, or with secured outdoor access (e.g. cat enclosures, secure gardens), should be the first‐line preventative strategy. Cats kept in this manner do not require FeLV vaccination.

Feline practitioners in Australia currently only have access to two inactivated pentavalent vaccines containing FeLV antigen (Fel‐O‐Vax® 5 and Fevac® 5) and lack access to any monovalent FeLV vaccine.

This limits flexibility to individualise vaccine protocols in Australia, preventing the use of a modified‐live core vaccine alongside a separate monovalent FeLV regimen, thereby making it more difficult for clinicians to follow published vaccination guidelines. This is disappointing and far from ideal.

We recommend vaccinating cats against FeLV using one of these pentavalent vaccines if a genuine risk of FeLV exposure has been demonstrated; for example, any in‐contact cats when FeLV is diagnosed in a multicat household.

We strongly advocate for the reintroduction of a monovalent FeLV vaccine in Australia; if one were to become available in Australia, we would likely expand our FeLV vaccination recommendation to be “Core” for all at‐risk cats.

No FeLV vaccine of any type is currently available in New Zealand, and therefore, we cannot make any meaningful FeLV vaccine recommendations to New Zealand veterinarians. We posit that New Zealand needs the reintroduction of at least one monovalent FeLV vaccine to give veterinarians the opportunity to vaccinate against FeLV infection, when justified.

### Testing, identification and separation approaches

Testing, identification of infected cats and separation approaches have contributed substantially to the reduction in prevalence of FeLV infection in some countries. Increasingly accurate and convenient diagnostic tests for detection of FeLV‐infected cats became available from the early 1980s.^74^ At that time, FeLV vaccines were not yet available. Soon after such tests became available, cat breeders in some countries introduced “test and removal” programmes. Later, some cat shelters also introduced such programmes. Some of these programmes were highly effective in reducing FeLV prevalence and may have led to eradication of FeLV infection from pet cats in a few countries. In situations when a single positive PoC test result was relied on for a diagnosis, undoubtedly some FeLV‐uninfected cats would have falsely tested FeLV‐positive and been euthanased unnecessarily during these eradication efforts.^75^ In the Netherlands, cat breeders were compelled to take part in these “test and removal” programmes.^76^

It may be possible to reduce substantially or eradicate FeLV infection at a regional or national level if concerted testing, identification and segregation efforts are made, although this may be more difficult if FeLV infection is present in the feral cat population, with transmission to client‐owned cats via outdoor access.^77^

### Keeping cats indoors

As with FIV, ensuring that kittens and cats are not exposed to FeLV is a sure way of preventing infection. It is of course vital to test all newly introduced cats to ensure they are FeLV‐negative. As well as close “friendly” contact with the saliva of FeLV‐infected cats leading to viral transmission, bites acquired by cats that are allowed access to the outdoors (often males) are an increasingly proposed mode of FeLV transmission.^21^, ^77^ Comingling FeLV‐infected and FeLV‐uninfected cats may be possible with FeLV vaccination of all in‐contact cats, particularly if there isn't a history of intercat aggression and wounding within the indoor social group.

Keeping pet cats 100% indoors, including secure outdoor enclosures, is the most practical way of preventing FeLV transmission from outside sources. It can also help prevent vehicular trauma, UV‐associated skin cancer, snake envenomation, tick paralysis and infection with FIV, while simultaneously minimising adverse impacts of pet cats on wildlife.^78^, ^79^ A survey of Australian and New Zealand cat owners in 2014–2015 found 34% of respondents classified their cats as indoor‐only, although over 60% of these owners stated that their cat had access to the outdoors at some point in their lives.^80^

**Figure 8:**
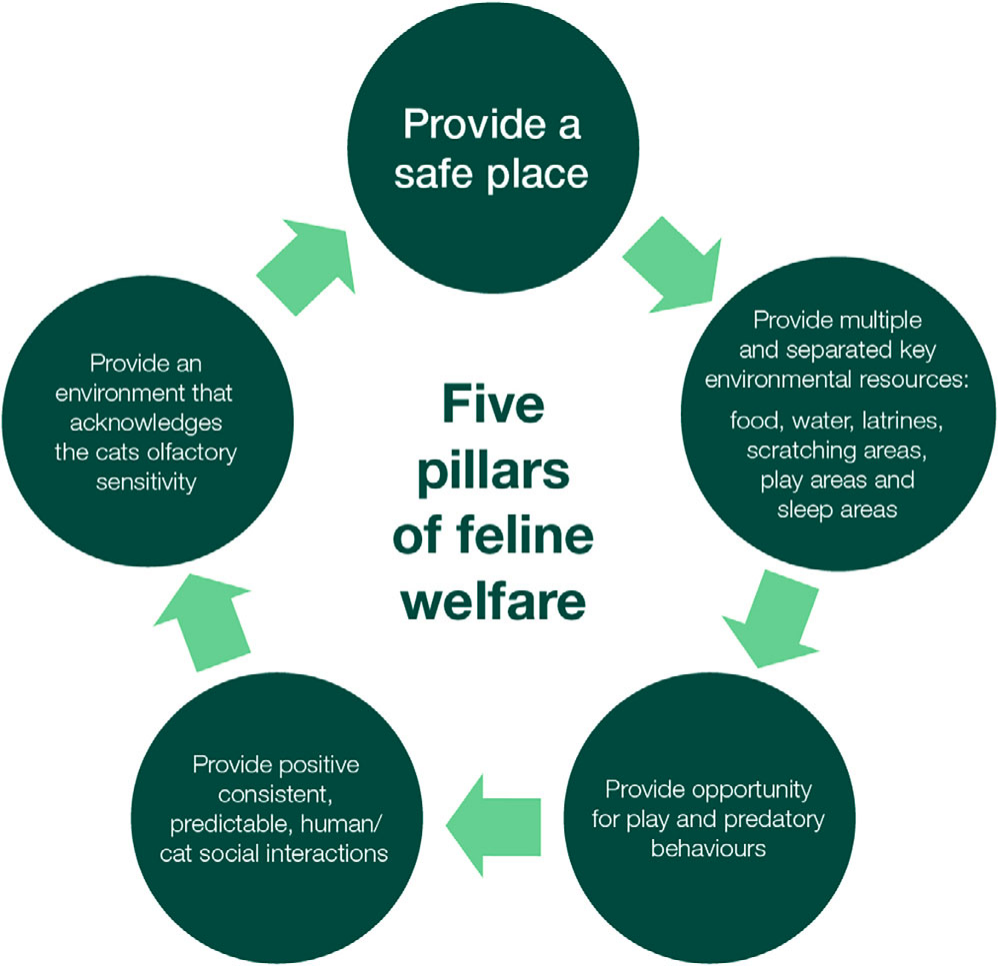
Keeping cats well, physically and emotionally, while housed entirely indoors requires fulfilling each of the five “pillars” for a healthy feline environment. Adapted from the 2013 American Association of Feline Practitioners (AAFP) and International Society of Feline Medicine (ISFM) feline environmental needs guidelines.^81^

Meeting the welfare needs of cats kept entirely indoors is vital. Familiarity with the American Association of Feline Practitioners (AAFP; now the Feline Veterinary Medical Association) and the International Society of Feline Medicine (ISFM; now International Cat Care) Feline Environmental Needs Guidelines may help when advising and encouraging clients who may be weighing up whether to keep their pet cat(s) 100% indoors, or indoors with access to an outdoor secured enclosure. Their “Five Pillars for a Healthy Feline Environment” are helpful.^81^ We believe this is the cornerstone of FeLV prevention (i.e., superior to vaccination) and is the best way to ensure longevity for owned cats (Figure 8).

Not all clients can manage to house their cat(s) 100% indoors, while completely addressing their welfare requirements.^80^ Consequently, FeLV vaccination may need to be considered in these cats.

### FeLV vaccines in Australia

Two inactivated pentavalent vaccines containing FeLV antigen are currently available in Australia. Fel‐O‐Vax® 5 is a pentavalent killed vaccine containing feline panleukopenia virus (FPV), feline calicivirus (FCV), feline herpesvirus type 1 (FHV‐1), *Chlamydia felis* and inactivated whole‐FeLV (initially manufactured by Fort Dodge, IA, USA, and later Boehringer Ingelheim Animal Health). Fevac® 5, a second pentavalent killed vaccine containing inactivated whole‐FeLV, is also distributed in Australia (Zoetis Animal Health, Rhodes, Sydney, Australia). FeLV vaccination in Australia initially requires two doses of one of these vaccines, and the manufacturer recommends annual revaccination with a single dose of the same vaccine.

In multiple studies, these two pentavalent vaccines have been reported to have a “preventable fraction” (efficacy) against FeLV ranging from 86% to 100% in experimental challenge infections, using small numbers of specific pathogen‐free (SPF) cats.^10^, ^82^, ^83^, ^84^, ^85^, ^86^, ^87^ One small study involving 20 cats reported an efficacy of only 44%.^41^ A higher percentage denotes better protection by the vaccine against FeLV challenge.

Vaccination does not completely remove the risk of FeLV transmission, since no FeLV vaccine is 100% protective. Furthermore, FeLV vaccination before experimental challenge was unable to completely prevent the development of regressive infections.^40^ Surprisingly, no field efficacy studies have been conducted on any FeLV vaccine anywhere in the world. Further background information on other FeLV vaccines previously available in Australia is provided in the Supplementary Materials (S3).

### FeLV vaccination recommendations in Australia

For a variety of reasons, including limited vaccine choice and perceived absence of justification, FeLV vaccination is not widely practiced in Australia, even in kittens and young cats. It is estimated only 2% of Australian pet cats are vaccinated against FeLV.^51^ The continuing low prevalence of progressive FeLV disease in Australia coincides with the fact that we do not really understand why FeLV has become rare in Australia compared with other countries such as Thailand or Brazil^32^, ^35^, ^88^, ^89^, ^90^, ^91^, ^92^; was it in part due to vaccination, or not? More local prevalence data are required in Australia to allow clinicians to make evidence‐based decisions about FeLV vaccination in their clinics. This could be undertaken by individual practices to inform on vaccine‐related decision‐making in a given setting.

FeLV vaccination should not be performed unnecessarily: it is not advised in areas without demonstrated FeLV infection, for cats housed strictly indoors, or in shelters practicing individual housing. We advise that cats should only be vaccinated against FeLV if a genuine risk of FeLV exposure has been demonstrated. For example, uninfected cats living group‐housed (where separation of cats is not possible) with progressively infected cats or FeLV‐infected cats in which the status (i.e., progressive vs. regressive) has not yet been determined, and where FeLV is endemic or known to be present. FeLV outbreaks, such as those reported in Sydney rescue facilities with group‐housed cats, highlight this need.^51^

In multiple cat households, when one cat is diagnosed with FeLV infection, all in‐contact cats should immediately be separated, tested and vaccinated against FeLV if they test negative and be vaccinated again 4 weeks later. Two weeks after completion of this primary course of FeLV vaccines, cats can be reunited.

If a monovalent FeLV vaccine becomes available again in Australia, we would likely align with the 2020 American Animal Hospital Association (AAHA)/AAFP and the 2024 World Small Animal Veterinary Association (WSAVA) Vaccination Guidelines. Both of these guidelines recommend FeLV vaccination as “Core” for all at‐risk cats, with at‐risk cats defined as juvenile and young adult cats <1 year of age with outdoor access in areas where FeLV is prevalent, after a locally informed risk–benefit assessment.^93^, ^94^ Monovalent FeLV vaccine availability would allow administration of a modified‐live core vaccine (for example against FPV in a shelter or during an outbreak) alongside a separate monovalent FeLV regimen. Vaccinating kittens twice with a monovalent FeLV vaccine, providing a booster 12 months later and then again every 2–3 years, is likely sufficient for most cats, as their susceptibility to infection decreases with age.^12^, ^95^

### FeLV vaccines and FeLV vaccination recommendations in New Zealand

There is an urgent need to investigate the current rates of progressive, regressive and abortive infections in representative cohorts of New Zealand cats and to determine the prevalence of progressive and regressive FeLV infections in New Zealand cats with lymphoma, to better assess the potential benefits of FeLV vaccination in New Zealand.

Given that FeLV infection is still present in New Zealand, we strongly advocate for the reintroduction of at least one monovalent FeLV vaccine. Further background information on FeLV vaccines previously available in New Zealand is provided as Supplementary materials (S3).

### Feline injection‐site sarcomas (FISS)

The most serious documented risk of vaccination (and other injections) in cats is FISS formation. This is a very rare, malignant neoplasm thought to be attributable to injection of vaccines and other substances. It was first described in the United States and reported shortly thereafter in Australia.^96^, ^97^ It is thought to be much less prevalent in Australia and New Zealand than the United States, in part due to absence of rabies vaccination, but epidemiological data from Australian and New Zealand are lacking. Nevertheless, the consequences for affected cats are catastrophic.

Given the apparent rarity of these tumors in Australia and New Zealand, we do not currently encourage distal limb or tail vaccination. However, avoiding the interscapular furrow and injecting approximately 4 cm lateral to the dorsal midline into the subcutis, over the convexity of the muscles covering the scapular spine, would allow earlier detection and diagnosis of any mass subsequent to vaccination. Although the role of adjuvants in the pathogenesis of FISS is unresolved,^98^ we suggest varying the site of injection of adjuvanted vaccines from vaccination to vaccination (i.e., left side one‐time, right side the next). The site of injection of each vaccine should be noted in the medical record as standard practice.

### Housing and hygiene in veterinary hospitals, shelters and catteries

Progressively FeLV‐infected cats must be physically separated from other cats. They should not be placed in the isolation ward or area with other cats that may have (for example) a contagious respiratory or gastrointestinal infection or a dermatophyte infection, because FeLV‐infected cats should be considered to be immunocompromised and potentially at increased risk for acquiring contagious infectious diseases. They can be housed in a normal cage in the hospital as they pose little or no risk of contagion via aerosol. FeLV is a fragile, enveloped virus that is very easily inactivated by detergents (including soap) and common disinfectants used in hospitals. It cannot survive in the environment for more than a few minutes. Routine hand hygiene (ideally wearing gloves) for staff before and after working with progressively FeLV‐infected cats, thorough cleaning and disinfection of cages and tables, and avoidance of sharing equipment and instruments that may become contaminated by the virus are all important. Dental instruments, syringes or surgical equipment should never be used on more than one cat without sterilisation between procedures. Regressively FeLV‐infected cats can be managed in hospitals and catteries in the same way as FeLV‐uninfected cats.^19^ Because FeLV is quite labile, staff are highly unlikely to bring sufficient quantities of the virus home on fomites to infect their own pet cats.

Haematogenous spread of FeLV can be avoided by routine screening of all potential blood donors with both p27 antigen testing and proviral DNA PCR testing (Table 3).^99^

## Management and treatment of FeLV‐infected cats

Critically, cats should not be euthanased because of a positive FeLV diagnosis. Some progressively infected cats will remain healthy for several years, and some will live longer than originally assumed.

FeLV infections should be managed as a chronic disease. Regular wellness veterinary care and good husbandry can help some FeLV‐infected cats continue to live good, quality lives.

No cure currently exists for progressive or regressive FeLV infection.

At this time, we suggest there is insufficient evidence to justify the use of any antiviral drugs for the treatment of FeLV infection.

Clinicians should be aware of products being marketed for the treatment of FeLV infection that lack scientific evidence of efficacy and safety.

Cats with abortive or regressive FeLV infections should be assumed to have the same status as an uninfected cat and are likely to have a normal lifespan. There may be a risk for late development of lymphoma, but currently there is not enough evidence to change management plans. Specific therapy for these cats, therefore, is not recommended, nor considered in these guidelines.^2^, ^6^, ^12^ We reiterate that regressively infected cats should never be used as blood donors due to the risk of iatrogenic FeLV transmission to blood product recipients.^22^, ^47^

**Figure 9:**
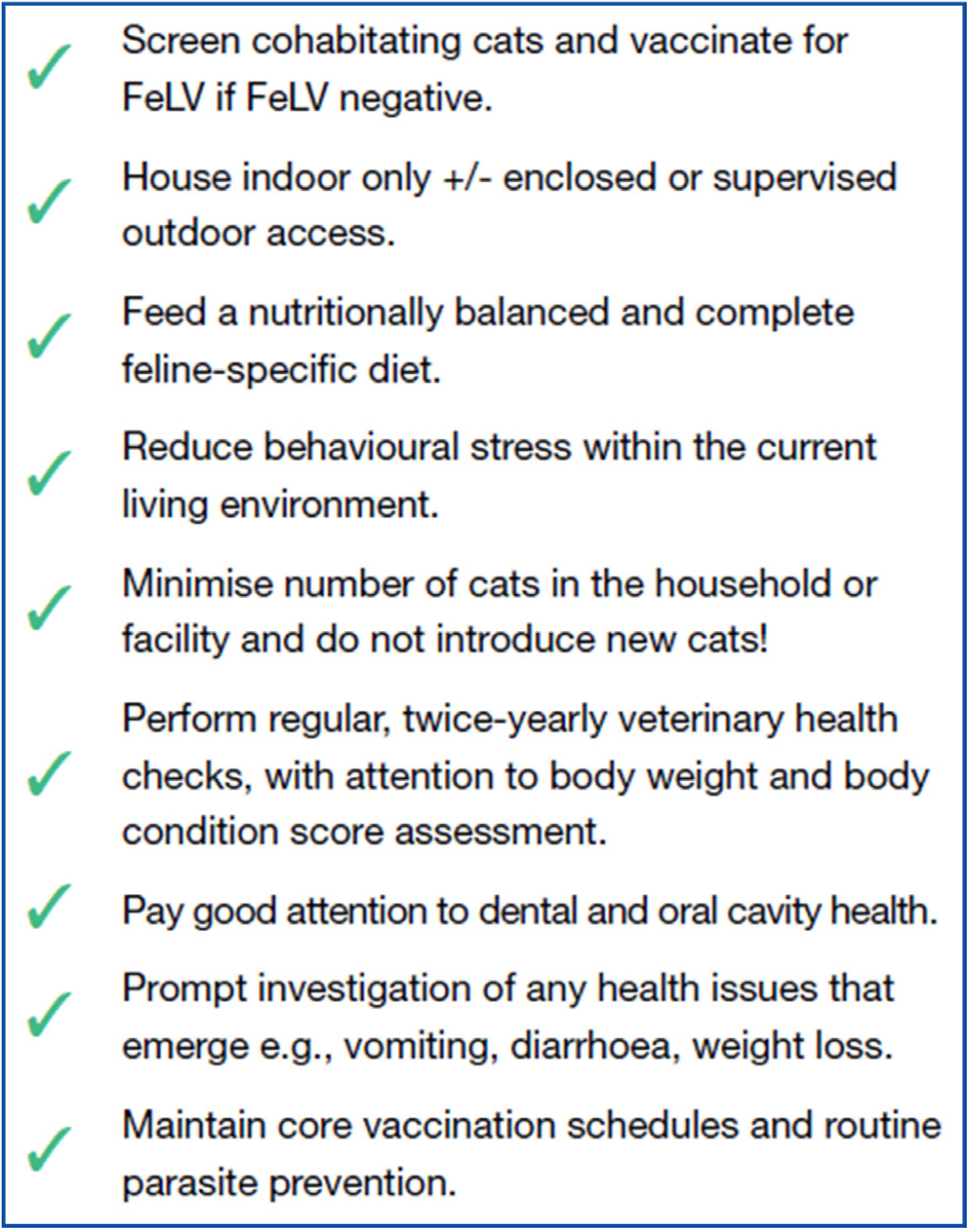
Checklist of health recommendations for basic health care and husbandry measures for all FeLV‐infected cats.

Cats with progressive FeLV infection are said to have up to a 90% mortality rate within 3 years of diagnosis.^6^, ^12^, ^19^ This is not only well‐known published evidence, but our clinical experience in Australia.^30^ Some progressively infected cats, however, will continue to live a healthy and happy life for many years, if well cared for. It is likely these cats have reduced FeLV viral loads for some reason.^6^, ^7^, ^12^ Although there is currently no highly effective antiviral treatment for FeLV infection, quality of life will be optimised if an appropriate health management plan is implemented promptly after diagnosis. Similar to recommendations given for the management of FIV‐infected patients, we advise that the key for FeLV‐infected patients is to do the basics well and to stay vigilant for comorbid disease processes (Figure 9).^1^ Diseases that would be considered likely or even possible in cats without FeLV infection should be excluded via thorough clinical investigation before ascribing any clinical problem to the retrovirus itself.

Routine core vaccinations should continue to be administered since FeLV‐infected cats can develop severe FPV‐related disease and upper respiratory tract infectious disease. Based on current evidence, we advise that both modified‐live and inactivated (killed) core vaccines can be administered safely and effectively to healthy‐appearing FeLV‐infected cats^19^, ^100^ and advise that preferably a licensed biennial or triennial core vaccine should be administered.^93^

### Monitoring for immunodeficiency in FeLV‐infected cats

A complete blood count can be performed on FeLV‐infected cats at the time of diagnosis and annually thereafter to monitor for haematological abnormalities such as leukaemia, thrombocytopenia and anaemia.^101^, ^102^, ^103^ Without any currently available effective antiviral treatments for FeLV infection, and the knowledge that every veterinary visit to collect blood will cause stress and expose possibly immunocompromised cats to other pathogens, this intervention seems excessive and difficult to justify.

Although routine monitoring of immune parameters (e.g., CD4+ and CD8+ lymphocyte counts and the CD4+/CD8+ ratio) has been reported for cats naturally infected with FeLV, it is not currently widely used. Recently, a review of three leukocyte ratios (neutrophil‐lymphocyte, monocyte‐lymphocyte and platelet‐lymphocyte) did not identify any that had useful prognostic value in retrovirus‐positive cats.^104^

### Antiretroviral and immunomodulatory agents: Which drugs might we consider for treating FeLV?

Theoretically, antiviral treatment of FeLV should be feasible using multimodal (combination) therapy. The types of drugs used to treat human immunodeficiency virus (HIV) infection in people likely should work in FeLV‐infected cats, but little evidence is available. We are not aware of any feline clinicians or internists in Australia who currently use specific antiretroviral therapy to manage cats with FeLV infections. At this stage, therefore, we suggest that any drug treatment of FeLV infection is best considered experimental.

We have included in the Supplementary Materials (S4) additional information pertaining to the agents that have been trialled in FeLV‐infected cats. To summarise, we do not recommend the use of the reverse transcriptase inhibitor zidovudine (also known as azidothymidine, AZT) due to poor treatment efficacy in naturally infected cats and the risk of bone marrow suppression, sometimes resulting in severe non‐regenerative anaemia. The integrase inhibitor raltegravir, although safe, requires further trials to demonstrate its long‐term efficacy on viral load and longevity. Recombinant feline interferon‐omega (rFeIFN‐ω) and recombinant human interferon‐alpha (rHuIFN‐α) cannot be recommended due to inconclusive efficacy and the treatment rebound effect that occurs when the medication is stopped, implying that lifelong, expensive therapy may be required. The recombinant chimaeric protein (RetroMAD1™) cannot be recommended due to an objective lack of demonstrated efficacy and convincing impact on longevity.

In the case series in Australia, 16 progressively FeLV‐infected cats were treated with different antiviral combinations. Six cats were administered RetroMAD1™ only (0.5 mg/kg orally twice daily), three cats were administered raltegravir only (10–15 mg/kg orally twice daily), three cats were administered RetroMAD1™ and raltegravir concurrently and four cats were administered raltegravir and AZT (5 mg/kg orally twice daily) concurrently. FeLV RNA and p27 antigen loads were measured at two timepoints (T1‐2 and T3‐5 months) during therapy and compared with baseline (pretreatment) levels to assess the response to therapy. Median survival time from commencement of FeLV treatment to death was also examined. The changes observed with treatment were biologically insignificant and overall were considered treatment failures according to HIV management guidelines. It was concluded from this small case series that results did not provide convincing support for the use of RetroMAD1™, raltegravir or AZT, alone or in combination, for the treatment of cats progressively infected with FeLV. Larger studies using this combination of drugs are not justified. It would be more fruitful to evaluate better drug candidates and drug combinations, drawn from HIV therapeutics.^30^

Since this study was published, the manufacturer of RetroMAD1™ has approached some of the authors (M.W. and R.M.) to suggest that the dose we used for our trial (which was recommended to us by the company at the time) was inadequate and recommended a dosage 2.4 times higher (1.2 mg/kg TID instead of 0.5 mg/kg BID).

In conclusion, further research, including preliminary cell culture studies to identify safe and effective alternative drug candidates with activity against FeLV, are clearly required. Technology exists now to screen thousands of potential agents against pathogens like FeLV for a relatively small cost. Once drug candidates have been identified, antiviral treatment trials would be best undertaken in countries or regions with high FeLV prevalences. We have heard anecdotally that stem cell therapy might be useful for treating FeLV infection, but more data are required. The new human drug lenacapavir, first FDA‐approved HIV capsid inhibitor, is given by subcutaneous injection every 6 months.^105^ If it has some efficacy against the capsid assembly of FeLV, it might be a very worthwhile drug to investigate for therapy, but as FeLV is a *Gammaretrovirus*, whereas HIV is a *Lentivirus*, one cannot assume efficacy without in vivo and in vitro studies. There are many other potential human antiretroviral drugs which could be evaluated similarly.

A future hope for FeLV treatment may reside in CRISPR/SaCas9‐assisted gene therapy. The theory is that, with lowering of viral loads in progressively infected cats using gene therapy, the cat's immune system may then be able to overcome the infection and direct it towards a regressive outcome. One study has demonstrated that this technology can reduce FeLV proviral load in vitro.^106^

### Neoplasia and chemotherapy treatment for FeLV‐infected cats

The important question for clinicians is, should chemotherapy treatment be considered in FeLV‐infected cats with lymphoma?

Overall, despite some conflicting reports, we advise that treating FeLV‐infected cats presenting with lymphoma with sequential multiagent chemotherapy is warranted, so long as such treatment interventions translate to improved quality of life for the individual.

FeLV‐infected cats are 62 times more likely to develop lymphoma or leukaemia compared with their noninfected counterparts.^34^ In contrast to FIV, a direct causal relationship has been established in relation to the development of neoplasia in FeLV‐infected cats,^3^ with FeLV reported to be causally associated with B‐ and T‐cell lymphomagenesis.^42^, ^43^

Many studies have shown that FeLV‐infected cats with progressive infections have a poorer prognosis with chemotherapy for lymphoma than FeLV‐uninfected cats, including reduced median survival time.^107^, ^108^, ^109^ That was the dogma until recently, and also the experience of several of the authors. A 2020 prospective study in Brazil assessed the efficacy of lomustine, vincristine, prednisolone and doxorubicin (LOPH) therapy in 19 FeLV‐infected client‐owned cats presenting with high‐grade multicentric or mediastinal lymphoma. A median survival time of 171 days was reported.^110^ Strikingly different was a 2022 study in Thailand examining cyclophosphamide, vincristine and prednisolone (COP) therapy in 92 FeLV‐infected cats with mediastinal lymphoma (and/or other anatomical sites of lymphoma). COP therapy was well tolerated and produced a median survival time of 338 days, with cats <4 years of age having longer survival times than cats aged >4 years.^111^ This reported median survival time for FeLV‐infected cats in these recent studies was comparable with, or superior to, survival times reported in non‐FeLV‐infected cats with lymphoma given chemotherapy.^107^, ^108^, ^109^

### Management of anaemia in FeLV‐infected cats

It is important for clinicians to consider the ethical implications of using donor cats' blood in a palliative care context.

It seems reasonable to administer a fresh typed blood transfusion to FeLV‐infected cats in the acute hospital setting to allow time for accurate diagnosis and response to ancillary and supportive interventions, but we have reservations concerning the routine use of transfusions to prolong life in FeLV‐infected cats.

The use of ‘donor’ to describe cats from which blood is taken is ethically contentious, given ‘consent’ is never given (by the cat) in a veterinary setting.

Anaemia, in many cases moderate to severe, is a common clinicopathologic abnormality in cats with progressive FeLV infection, as is thrombocytopenia.^35^, ^69^, ^102^ If thrombocytopenia is present, it may have implications with regard to the risk of blood loss contributing to anaemia, although reports of this are scarce.^112^ Anaemia is typically non‐regenerative but can be regenerative in a small percentage of cases.^3^, ^12^, ^69^, ^113^, ^114^ Macrocytosis (larger than normal erythrocytes) and/or increased anisocytosis are common features of FeLV‐associated non‐regenerative anaemia.^69^, ^115^ Pure red cell aplasia, characterised by markedly impaired bone marrow production of red blood cells related to infection with the FeLV‐C subtype, and aplastic anaemia (deficiency of all types of red and white blood cells, i.e., pancytopenia), associative immune‐mediated haemolytic anaemia (IMHA) and myeloproliferative diseases have all been reported. Anaemia of chronic inflammatory disease is also a possible contributor.^2^, ^69^, ^116^, ^117^, ^118^, ^119^, ^120^, ^121^, ^122^, ^123^, ^124^ Coinfection with haemotropic mycoplasmas should be considered, with lack of regeneration not precluding the possibility.^125^, ^126^

We strongly advise confirmation of any in‐house FeLV antigen positive result by proviral PCR testing, even in cats presenting with anaemia, as false‐positive results do occur in cats presenting with clinical signs consistent with FeLV infection.^66^, ^67^A complete blood count and reticulocyte count should be performed to confirm the presence or absence of erythrocyte regeneration. Repeat haematology is recommended after 5–7 days if a preregenerative response is possible (i.e., the anaemia has not been documented to be chronic). Infectious disease screening including PCR testing for feline haemotropic mycoplasmas is recommended,^127^ and saline agglutination and Coombs' testing should be considered. Bone marrow aspiration and core biopsy are indicated in the presence of bi‐ or pancytopenia, severe, persistent or progressive non‐regenerative anaemia and/or circulating atypical cells.^114^

We consider it reasonable to administer a fresh typed blood transfusion to unstable, weak or anorexic FeLV‐infected anaemic cats in order to stabilise them while diagnostics are pending, and any therapeutic interventions are planned and/or commenced. Repeated blood transfusions in cats with progressive FeLV infection are contentious, taking into consideration the high value of fresh blood products, the potential to cause harm to a donor cat (sedation is often required), the requirement to avoid rebleeding the same donor cat frequently (therefore risking being unable to utilise the donor again if a cat with a curable disease process presents during this period), and the guarded prognosis associated with FeLV‐associated non‐regenerative anaemia.

Administration of erythropoiesis‐stimulating agents such as darbepoetin (starting dose 1.0 μg/kg once weekly in conjunction with iron dextran 10 mg/kg IM q3‐4 weeks) has received mention for use in cats with non‐regenerative anaemia unrelated to chronic kidney disease (CKD), for which it is more clearly indicated.^114^, ^128^ Endogenous erythropoietin levels are elevated in cats with FeLV‐related anaemia, thus this treatment would appear unlikely to be efficacious, and the authors are unaware of any reports of successful use in this scenario.^123^, ^129^ Recently, daily molidustat treatment significantly increased the haematocrit in cats with CKD‐associated anaemia^130^; it could be investigated for use in the context of FeLV‐associated anaemia. If haemotropic mycoplasma infection is suspected or confirmed, treatment is typically with doxycycline monohydrate (5 mg/kg BID or 10 mg/kg SID; courses of 2 weeks are usually recommended, although some have suggested longer treatment courses of up to 6 weeks increase the likelihood of eliminating infection; all doses should be followed with food or water to avoid oesophagitis).^127^

Administration of immunosuppressive medications including prednisolone may be considered in FeLV‐infected cats with suspected regenerative or non‐regenerative IMHA,^122^, ^131^ but the impact on the concurrent FeLV infection *per se* is unknown and possibly deleterious. Although the median survival time is short (1–2 months depending on the form), some cats with myelodysplasia and FeLV can also survive for extended periods with administration of prednisolone and/or cytarabine.^120^ Finally, administration of rHuIFN‐α or rFeIFN‐ω, as previously mentioned, may increase the red cell counts of cats naturally infected with FeLV during the period of administration and thus may be helpful.^132^, ^133^ Eltrombopag and sirolimus are now being used in human patients with pure red cell aplasia and might provide useful for FeLV‐associated anaemia and cytopenias, although neither has been trialled in cats to date.^134^, ^135^

## Conclusion and future priorities

Much has changed with regard to the diagnosis and classification of FeLV infections over the past 20 years. This review updates Australian and New Zealand clinicians on recent advances and summarises current knowledge and approaches to FeLV infection. As well as progressive infections, FeLV exposure often results in abortive, localised (focal) or regressive infections. We suspect that low grade FeLV exposure and infection may be much more common than many Australian and New Zealand veterinarians realise. More practical research addressing the prevalence of FeLV infection in Australia and New Zealand, and antiviral treatment options, is needed, although the impetus for such work is diminished by the low incidence rate for progressive FeLV disease in our countries. Although we may not yet know how to treat FeLV infection effectively, we certainly know how to prevent it, and FeLV vaccination of at‐risk kittens and young adult cats remains important.

## Conflicts of interest and sources of funding

Boehringer Ingelheim Animal Health Australia, supplier of the Fel‐O‐Vax® 5 vaccine in Australia, provided an honorarium to all listed authors to create a documented titled “Australian Feline Retrovirus Management Guidelines: Part 2 Feline Leukaemia Virus (FeLV)”. That document heavily informed the writing of this manuscript. Boehringer Ingelheim did not ask the authors to publish this document in a peer‐reviewed journal. It was entirely an independent decision of the authors listed.

## Supporting information


**Supplementary Material S1.** FeLV prevalence (Section 2).

**Supplementary Material S2.** FeLV testing (Section 3).

**Supplementary Material S3.** FeLV vaccination (Section 4).

**Supplementary Material S4.** FeLV treatment (Section 5).

**Table S1.** A summary of prices for FeLV testing available to veterinarians in Australia. Prices correct at the time of writing. FIV, feline immunodeficiency virus; NSW, New South Wales; PCR, polymerase chain reaction; PoC, point‐of‐care.

**Table S2.** Summary of test results for the different categories of FeLV infection. Neutralising antibody (NAb) testing is currently not available to clinicians in Australia or New Zealand to identify abortive infections.

## Data Availability

The data that support the findings of this study are available on request from the corresponding author. The data are not publicly available due to privacy or ethical restrictions.

## Footnotes

^i^
The study was supported by the University of Florida, Maddie's Fund, Winn Feline Foundation (EveryCat Health Foundation), and Austin Pets Alive!.

^ii^
Australia ‐ IDEXX, East Brisbane, Queensland; Veterinary Pathology Diagnostic Services, University of Sydney, Sydney, NSW; Gribbles Veterinary Pathology, Glenside, South Australia; Vetnostics, Macquarie Park, NSW. New Zealand ‐ Awanui Veterinary, Palmerston North; IDEXX, Palmerston North.
