# Supplementary material for: Feline leukaemia virus (FeLV) infection in domestic pet cats in Australia and New Zealand: Guidelines for diagnosis, prevention and management
Source: Aust Vet J. 2025 Jul 26;103(10):617–35. doi: 10.1111/avj.13470 (PMC12500364; doi:10.1111/avj.13470)
Supplement: Supplementary file 1 — Supplementary Material S1. FeLV prevalence (Section 2). [file AVJ-103-617-s001.docx]

***Prevalence of progressive FeLV infection in Australia: Additional historical context and unpublished results***

Although systematic prevalence studies of infectious diseases such as FeLV were rare or non-existent prior to the 1990s, FeLV infection was considered common in Australia in the 1970s and early 1980s. Widespread screening of catteries and other feline populations in Australia for FeLV did not occur, but for whatever reasons, the prevalence of FeLV in the eastern Australian states was subsequently considered low to extremely low from the mid-1990s, before any FeLV vaccine was commercially available in Australia (Leucogen® FeLV, Virbac Animal Health, Carros, France, in 1998) [1].

In a representative sample of 200 healthy pet cats collected in Sydney in 1994-1995, FeLV antigenaemia was uncommon (<2%). In the same study, samples submitted by city and country practitioners in New South Wales (NSW) to a private clinical pathology laboratory in Sydney yielded 1.4% (11/761) positivity using the same laboratory-based antigen test; approximately 88% of these samples were from ‘sick’ cats [2]. This observation (~2% prevalence) was confirmed in 340 samples collected from pet cats in Sydney in 2002-2004 with PoC testing [3,4]. Both studies were conducted before polymerase chain reaction (PCR) testing for FeLV was readily available for test confirmation and detection of regressive infections.

In 2021, opportunistic PoC testing at a clinic in Wagga Wagga, NSW (a relatively large regional city) with Anigen Rapid® FeLV kits produced 4.5% (2/44) FeLV antigen-positive results, but confirmatory FeLV PCR testing for proviral DNA was not performed. One antigen-positive cat was a young adult stray cat that presented underweight and with lethargy and diarrhoea (euthanased the same day as FeLV testing), while the other antigen-positive cat was an in-contact healthy 2.8-year-old cat that was euthanased 1.5 years after FeLV diagnosis due to a non-regenerative anaemia (unpublished data).

The prevalence of progressive FeLV infection in cats presenting with lymphoma in Australia has also declined over time. Of lymphoma samples collected at a veterinary teaching hospital in Sydney between 1967 and 1972 and examined for FeLV using electron microscopy, 9/11 (82%) cases were confirmed to be infected. Transmission experiments then showed that macerated tissue extracts from cats with lymphoma, when injected into normal healthy kittens, resulted in the rapid development of lymphoma in the challenged kittens [5].

In a study of 60 cats with lymphoma seen in the same institution from 1984 to 1994 (i.e. approximately 20 years later), lymphoma appeared predominantly as a disease of older cats and only 2/22 cats (9%) tested positive to FeLV antigen with an ELISA [6]. In a larger and more systematic study conducted from 1995 to 1998, only 2/107 cats (2%) with lymphoma had progressive FeLV infections on the basis of PoC testing using serum [7].

***Prevalence of progressive FeLV infection in New Zealand: Additional historical context***

Testing for FeLV in NZ was not available before 1980. On samples collected in 1978–1979, immunofluorescent antibody (IFA) testing performed in the USA found an overall prevalence of progressive FeLV infection of 11% (26/230). The prevalence of FeLV infection in multi-cat households was higher (16%) than in single-cat households (3%), and infection was found in a mixture of healthy and sick cats, including some with lymphoma [8]. Soon afterwards, based on samples collected in 1980–1981, the prevalence of progressive FeLV infection in cats tested by a commercial PoC kit using blood was 4% (13/293) [8]. In 1982–1983, the prevalence of FeLV in NZ was 6.5% (30/465); the slightly higher prevalence reported was perhaps due to bias from more multi-cat households being tested [9].

***Prevalence of regressive FeLV infection in Australia***

One of the first pointers to the importance of appreciating regressive infections as a key to understanding of the overall epidemiology of FeLV-related disease was a study of large cell lymphoma in Australian cats. In this study, a substantial proportion of lymphomas (21/86; 24%) contained FeLV proviral DNA, but only 2% of these tumour-bearing cats had detectable FeLV antigen in their serum. Thus, the vast majority of lymphoma-associated FeLV infections were ‘regressive’. These cats were indistinguishable in their response to chemotherapy compared to FeLV-negative cats, whereas FeLV-infected cats with progressive disease died quickly despite multiagent sequential chemotherapy [7]. More recent Australian feline lymphoma research yielded similar findings (Supplementary Table 1) [10].

**Supplementary Table 1.** Prevalence of progressive and regressive FeLV infections in cases of lymphoma. Results from Australian research are shown in bold text.

|  | Canada 1993 [11] | Australia 2001 [7] | Australia 2018 [10] |
| --- | --- | --- | --- |
| Prevalence of progressive infections in lymphoma cases | 47.5% (19/40) | **1.9%** (2/107) | **3.3%** (4/122) |
| Prevalence of regressive infections in lymphoma cases | 17.5% (7/40) | **22.1%** (19/86) | **26.2%** (11/42) |

In 2009, no regressive infections were detected in healthy, young (mean age 3 months; all < 1 year), client-owned cats in Sydney, most acquired from rescue societies (0/169) [12].

Regressive infections were identified in 2% (9/440) of healthy pet cats in Australia two years-of-age or older with some level of outdoor access sampled during 2013-2015 (i.e., 4.5 times more common than progressive infections in the same cohort). This result indicated that FeLV exposure and infection might be more common in the general pet population in Australia than previously appreciated [13].

In two FeLV outbreaks reported in Sydney-based rescue facilities, a combined prevalence of regressive infections of 25% (22/89) was reported. The ratio of regressive to progressive infections in these cohorts was 1–1.25, suggesting that progressive infections are more likely than regressive infections in multi-cat situations compared to the general pet cat population [13,14]. This is probably a consequence of a combination of factors including higher virus exposure (higher number of FeLV-infected cats), higher infective doses, co-infections, and higher stress levels resulting from the proximity to numerous other cats [15].

***Prevalence of regressive FeLV infection in New Zealand***

No studies have been performed to date to report the prevalence of regressive infections in NZ, which therefore represents a substantial knowledge gap. Investigation of the prevalence of regressive infections in cats with lymphoma, and the general cat population in NZ, are needed.

***Prevalence of abortive FeLV infection in Australia***

Abortive infections were identified in 11% (47/440) of healthy pet cats in Australia two years-of-age or older with some level of outdoor access. This meant abortive infections were the most common consequence of FeLV exposure in Australian pet cats, 23.5 times more common than progressive infections and 4.3 times more common than progressive and regressive infections combined [13].

***Prevalence of abortive FeLV infection in New Zealand***

No studies have been performed to date to report the prevalence of abortive infections in NZ. This work is needed to help NZ veterinarians to understand the risk of FeLV exposure to cats in their care.

**References**

1. Jarrett, O.; Ganière, J.-P., Comparative studies of the efficacy of a recombinant feline leukaemia virus vaccine. *Vet. Rec.* **1996,** 138, (1), 7-11.

2. Malik, R.; Kendall, K.; Cridland, J.; Coulston, S.; Stuart, A.; Snow, D.; Love, D., Prevalences of feline leukaemia virus and feline immunodeficiency virus infections in cats in Sydney. *Aust. Vet. J.* **1997,** 75, (5), 323-327.

3. Norris, J. M.; Bell, E. T.; Hales, L.; Toribio, J. A.; White, J. D.; Wigney, D. I.; Baral, R. M.; Malik, R., Prevalence of feline immunodeficiency virus infection in domesticated and feral cats in eastern Australia. *J. Feline Med. Surg.* **2007,** 9, (4), 300-8.

4. Westman, M. E.; Paul, A.; Malik, R.; McDonagh, P.; Ward, M. P.; Hall, E.; Norris, J. M., Seroprevalence of feline immunodeficiency virus and feline leukaemia virus in Australia: Risk factors for infection and geographical influences (2011-2013). *J. Feline Med. Surg. Open Rep.* **2016,** 2, (1), 1-11.

5. Sabine, M.; Wright, R. G.; Love, D. N., Studies on feline lymphosarcoma in the Sydney area. *Aust. J. Exp. Biol. Med. Sci.* **1974,** 52, (2), 331-340.

6. Court, E. A.; Watson, A. D. J.; Peaston, A. E., Retrospective study of 60 cases of feline lymphosarcoma. *Aust. Vet. J.* **1997,** 75, (6), 424-427.

7. Gabor, L.; Jackson, M.; Trask, B.; Malik, R.; Canfield, P., Feline leukaemia virus status of Australian cats with lymphosarcoma. *Aust. Vet. J.* **2001,** 79, (7), 476-481.

8. Jones, B. R.; Lee, E. A., Feline leukaemia virus testing. *N.Zeal. Vet. J.* **1981,** 29, (10), 188-189.

9. Jones, B. R.; Lee, E. A.; Pauli, J. V., Feline leukaemia virus testing. *N.Zeal. Vet. J.* **1983,** 31, (8), 145-146.

10. McLuckie, A. J.; Barrs, V. R.; Lindsay, S.; Aghazadeh, M.; Sangster, C.; Beatty, J. A., Molecular diagnosis of Felis catus gammaherpesvirus 1 (FcaGHV1) infection in cats of known retrovirus status with and without lymphoma. *Viruses* **2018,** 10, (3).

11. Jackson, M.; Haines, D.; Meric, S.; Misra, V., Feline leukemia virus detection by immunohistochemistry and polymerase chain reaction in formalin-fixed, paraffin-embedded tumor tissue from cats with lymphosarcoma. *Can. J. Vet. Res.* **1993,** 57, (4), 269.

12. Beatty, J. A.; Tasker, S.; Jarrett, O.; Lam, A.; Gibson, S.; Noe-Nordberg, A.; Phillips, A.; Fawcett, A.; Barrs, V. R., Markers of feline leukaemia virus infection or exposure in cats from a region of low seroprevalence. *J. Feline Med. Surg.* **2011,** 13, (12), 927-33.

13. Westman, M.; Norris, J.; Malik, R.; Hofmann-Lehmann, R.; Harvey, A.; McLuckie, A.; Perkins, M.; Schofield, D.; Marcus, A.; McDonald, M.; Ward, M.; Hall, E.; Sheehy, P.; Hosie, M., The diagnosis of feline leukaemia virus (FeLV) infection in owned and group-housed rescue cats in Australia. *Viruses* **2019,** 11, (6), 503.

14. Gomes-Keller, M. A.; Gonczi, E.; Tandon, R.; Riondato, F.; Hofmann-Lehmann, R.; Meli, M. L.; Lutz, H., Detection of feline leukemia virus RNA in saliva from naturally infected cats and correlation of PCR results with those of current diagnostic methods. *J. Clin. Microbiol.* **2006,** 44, (3), 916-22.

15. Beczkowski, P. M.; Litster, A.; Lin, T. L.; Mellor, D. J.; Willett, B. J.; Hosie, M. J., Contrasting clinical outcomes in two cohorts of cats naturally infected with feline immunodeficiency virus (FIV). *Vet. Microbiol.* **2015,** 176, (1-2), 50-60.
