# Supplementary material for: Feline leukaemia virus (FeLV) infection in domestic pet cats in Australia and New Zealand: Guidelines for diagnosis, prevention and management
Source: Aust Vet J. 2025 Jul 26;103(10):617–35. doi: 10.1111/avj.13470 (PMC12500364; doi:10.1111/avj.13470)
Supplement: Supplementary file 2 — Supplementary Material S2. FeLV testing (Section 3). [file AVJ-103-617-s003.docx]

***Additional FeLV tests available to veterinarians outside of Australia and NZ***

In Australia and NZ, testing options for FeLV are limited compared with what is available in Europe and North America.

In Europe and North America, IDEXX use a laboratory-based ELISA for confirmatory p27 antigen testing following a positive SNAP® FeLV result (PetChek® FeLV 15). The test includes a neutralization step for confirmation of FeLV-specific antigen. A qualitative p27-positive result on the ELISA in the absence of detectable proviral DNA by FeLV Quant RealPCR^TM^ testing is interpreted as a ‘low positive’, thereby identifying cats with a low level of FeLV-specific antigen, which may be useful prognostically. Further information about classification of FeLV-infected cats into ‘high positives’ and ‘low positives’ based on the qualitative PetChek® FeLV 15 result and the quantitative FeLV Quant RealPCR^TM^ result, and the prognostic value of this classification scheme, is found in Table 2 and Figure 3 of this publication [1].

Immunofluorescent antibody (IFA) testing to detect p27 antigen in neutrophils and platelets, previously offered in Australia and NZ through Vetpath Laboratory Services (Vetnostics) and Massey University respectively, is no longer available in either country. Virus isolation (VI) and neutralising antibody (NAb) testing are also not available to clinicians in Australia or NZ. IFA, VI, and NAb testing are all available in other areas of the world including North America and Europe (NAb testing is available only in Scotland).

In some countries (e.g., Switzerland), FeLV PCR testing includes a reverse transcriptase (RT) step to also detect viral RNA in blood or saliva. None of the FeLV PCR tests available in Australia or NZ currently includes a RT step.

In some parts of Europe, RT-PCR testing to detect FeLV RNA in saliva is recommended and utilised since it is extremely accurate. FeLV can be detected in saliva as early as one week after FeLV exposure. Saliva RNA results generally are highly correlated with the detection of p27 antigen in the blood of infected cats (and therefore in turn are also a measure of viraemia), and when screening group-housed cats, pooling of samples (maximum of 10 samples) can make testing much more affordable than PoC testing of blood [2-6]. Of course, if a pooled sample tests RT-PCR positive, individual testing is then required to identify the specific FeLV-infected cat(s). RT-PCR testing of saliva to detect FeLV RNA is currently not available in Australia or NZ but may become available in the future.

**References**

1. Beall, M. J.; Buch, J.; Clark, G.; Estrada, M.; Rakitin, A.; Hamman, N. T.; Frenden, M. K.; Jefferson, E. P.; Amirian, E. S.; Levy, J. K., Feline leukemia virus p27 antigen concentration and proviral DNA load are associated with survival in naturally infected cats. *Viruses* **2021,** 13, (2), 302.

2. Hofmann-Lehmann, R.; Hartmann, K., Feline leukaemia virus infection: A practical approach to diagnosis. *J. Feline Med. Surg.* **2020,** 22, (9), 831-846.

3. Little, S.; Levy, J.; Hartmann, K.; Hofmann-Lehmann, R.; Hosie, M.; Olah, G.; Denis, K. S., 2020 AAFP feline retrovirus testing and management guidelines. *J. Feline Med. Surg.* **2020,** 22, (1), 5-30.

4. Hartmann, K.; Hofmann-Lehmann, R., What's new in feline leukemia virus infection. *Vet. Clin. North Am. Small Anim. Pract.* **2020,** 50, (5), 1013-1036.

5. Gomes-Keller, M. A.; Gonczi, E.; Tandon, R.; Riondato, F.; Hofmann-Lehmann, R.; Meli, M. L.; Lutz, H., Detection of feline leukemia virus RNA in saliva from naturally infected cats and correlation of PCR results with those of current diagnostic methods. *J. Clin. Microbiol.* **2006,** 44, (3), 916-22.

6. Gomes-Keller, M. A.; Tandon, R.; Gonczi, E.; Meli, M. L.; Hofmann-Lehmann, R.; Lutz, H., Shedding of feline leukemia virus RNA in saliva is a consistent feature in viremic cats. *Vet. Microbiol.* **2006,** 112, (1), 11-21.
