# Supplementary material for: Feline leukaemia virus (FeLV) infection in domestic pet cats in Australia and New Zealand: Guidelines for diagnosis, prevention and management
Source: Aust Vet J. 2025 Jul 26;103(10):617–35. doi: 10.1111/avj.13470 (PMC12500364; doi:10.1111/avj.13470)
Supplement: Supplementary file 3 — Supplementary Material S3. FeLV vaccination (Section 4). [file AVJ-103-617-s002.docx]

***FeLV vaccines in Australia: Additional historical context***

Australia once had up to four FeLV vaccines on the market. Fel-O-Vax® Lv-K (initially manufactured by Fort Dodge, IA, USA, and later Boehringer Ingelheim Animal Health) is a monovalent FeLV vaccine that contains the same FeLV antigen as Fel-O-Vax® 5 [1,2]. Leucogen® FeLV (Virbac Animal Health, Carros, France), is an adjuvanted, recombinant vaccine that contains p45 (the non-glycosylated form of the surface unit envelope glycoprotein gp70) derived from FeLV and expressed in *Escherichia coli* [3,4]. Leucogen® FeLV was the first FeLV vaccine released in Australia in 1998, with Fel-O-Vax® 5 and Fel-O-Vax® Lv-K released around 2000.

Unfortunately, the monovalent FeLV vaccines Fel-O-Vax® Lv-K and Leucogen® FeLV were both discontinued in Australia by the manufacturers in 2019-2020. Although Zoetis has an APVMA-registered monovalent FeLV vaccine, it is not currently commercially available in Australia. As a result, feline practitioners in Australia currently lack access to a monovalent FeLV vaccine.

***FeLV vaccines in New Zealand: Additional historical context***

Two FeLV vaccines were registered in NZ for use in the prevention of disease, with vaccine supply dating back to at least 2003. The available vaccines were Leukocell® 2 (Zoetis Animal Health), an inactivated vaccine containing gp70 from three different FeLV subtypes, and Leucogen® FeLV (Virbac Animal Health). Dwindling demand from veterinarians based on a perceived low FeLV prevalence resulted in both vaccines being removed from the market between 2015 and 2017 [5]. Consequently, no FeLV vaccine is currently available in NZ.

**References**

1. Scott, F. W., Feline infectious diseases. *Feline Practitioners Seminar Proceedings.* Cornell University, USA, July 26-29, 1996.

2. Westman, M.; Norris, J.; Malik, R.; Hofmann-Lehmann, R.; Parr, Y. A.; Armstrong, E.; McDonald, M.; Hall, E.; Sheehy, P.; Hosie, M. J., Anti-SU antibody responses in client-owned cats following vaccination against feline leukaemia virus with two inactivated whole-virus vaccines (Fel-O-Vax® Lv-K and Fel-O-Vax® 5). *Viruses* **2021,** 13, (2), 240.

3. Jarrett, O.; Ganière, J.-P., Comparative studies of the efficacy of a recombinant feline leukaemia virus vaccine. *Vet. Rec.* **1996,** 138, (1), 7-11.

4. Sparkes, A. H., Feline leukaemia virus: a review of immunity and vaccination. *J. Small Anim. Pract.* **1997,** 38, (5), 187-194.

5. Luckman, C.; Gates, M. C., Epidemiology and clinical outcomes of feline immunodeficiency virus and feline leukaemia virus in client-owned cats in New Zealand. *J. Feline Med. Surg. Open Rep.* **2017,** 3, (2), 1-9.
