# Supplementary material for: Feline leukaemia virus (FeLV) infection in domestic pet cats in Australia and New Zealand: Guidelines for diagnosis, prevention and management
Source: Aust Vet J. 2025 Jul 26;103(10):617–35. doi: 10.1111/avj.13470 (PMC12500364; doi:10.1111/avj.13470)
Supplement: Supplementary file 4 — Supplementary Material S4. FeLV treatment (Section 5). [file AVJ-103-617-s005.docx]

***Review of antiretroviral and immunomodulatory agents trialled for the treatment of FeLV-infected cats***

The reverse transcriptase inhibitor zidovudine (also known as azidothymidine; AZT) initially showed promise, inhibiting FeLV replication in vitro [1-6]. AZT also showed efficacy in vivo using experimental models, protecting against persistent viraemia and bone marrow infection when used less than one week after experimentally induced FeLV infection [6,7]. Unfortunately, these protective effects do not appear to extend to naturally acquired FeLV infection. In one study of 44 naturally FeLV-infected cats in Germany, of which 82% cats presented with stomatitis, treatment consisted of AZT (5 mg/kg BID orally) for six weeks with or without the addition of human interferon-α2a (10^5^ IU/kg SID administered subcutaneously). No significant improvement in stomatitis or blood parameters, including p27 antigen concentrations and immunological parameters, were observed in any treatment group, except for a more than doubling of the absolute CD4+ count in cats treated with both drugs [8].

AZT may also induce bone marrow suppression, sometimes resulting in a severe non-regenerative anaemia. Consequently, AZT is contraindicated in cats with existing bone marrow suppression. If AZT therapy is commenced, complete blood counts should be undertaken regularly (at least weekly for the first month), and treatment immediately discontinued if the haematocrit drops below 20%. Anaemia usually resolves within a few days with cessation of AZT therapy [9]. Thus, the overall therapeutic efficacy of AZT is even less promising in FeLV-infected cats than in FIV-infected cats, and AZT use is not recommended in these guidelines [10,11].

The integrase inhibitor raltegravir was shown to inhibit viral replication in vitro [12] and is well tolerated in cats. When administered orally twice daily to cats with experimentally induced FeLV infection (20 or 40 mg orally BID; equivalent to about 5-10 mg/kg BID), only small reductions of plasma viral load were observed (over the 4 month treatment period) and a rebound in plasma viral load occurred on discontinuation of the drug [13]. This implies that life-long therapy would probably be required, a cost prohibitive option for most clients. The drug must be compounded for feline use. There are no field studies that show that such therapy prolongs life, but at least it is well tolerated. It might be the first step in combination antiretroviral therapy.

Recombinant feline interferon-omega (rFeIFN-ω) is an immune-modulator that has demonstrated antiviral, antiproliferative, and immunomodulatory activities (e.g., against canine parvovirus). In a study, rFeIFN-ω was administered subcutaneously (1 million U/kg), once daily for 5 consecutive days, to naturally FeLV-infected cats with at least one clinical sign of disease. Three courses of treatment were administered over a 9-week period. Treatment resulted in a minor reduction in the severity of clinical signs (using a clinical scoring system) and significantly lower mortality in the 9 and 12 months following the 9-week treatment period in comparison to placebo controls (1.4 and 1.7 fold higher risk of death in controls, respectively) [14]. The supply of this drug can be erratic and it is expensive.

In another study, recombinant human interferon-alpha (rHuIFN-α) was administered orally (60 IU per cat), once daily in alternate weeks during a 4-month treatment period, for a total of 56 doses. This was an ultralow dose of human rHuIFN-α administered to cats designed to avoid the development of antibodies against human IFN [15]. Naturally infected cats were treated, 60% of which had clinical signs at the start of the study (half had mild disease and half had severe disease, based on a clinical scoring system). Similarly to treatment with feline IFN-ω, treatment with human IFN-α improved clinical signs during and following the 4-month treatment period, with an associated mild reduction in plasma viral loads [16,17]. Cats tolerated the treatment well and showed improvement in immune status (improved CD4+/CD8+ ratios). Unfortunately, rebounds in plasma antigenaemia, proviral DNA load and percentage of cats with anaemia, to name a few measurements, were observed on discontinuation of therapy, again implying that life-long, expensive therapy may be required.

A recombinant chimeric protein (RetroMAD1^TM^) with proposed three-pronged antiviral activity due to it comprising a trifecta of drugs (retrocyclin, MAP30 and dermaseptin) is commercially available in some countries for the treatment of infectious diseases including natural FeLV infection. One paper suggested that treatment with RetroMAD1^TM^ (0.4 mg/kg/day orally, divided equally and administered 12 hours apart) improved survival time of FeLV-infected cats, although viral loads were not measured [18]. This paper was published by the co-inventor of the patented drug combination.

**References**

1. Hoover, E. A.; Zeidner, N. S.; Mullins, J. I., Therapy of presymptomatic FeLV-induced immunodeficiency syndrome with AZT in combination with alpha interferon. *Ann. N. Y. Acad. Sci.* **1990,** 616, 258-269.

2. Mathes, L.; Polas, P.; Hayes, K.; Swenson, C.; Johnson, S.; Kociba, G., Pre-and postexposure chemoprophylaxis: Evidence that 3'-azido-3'-dideoxythymidine inhibits feline leukemia virus disease by a drug-induced vaccine response. *Antimicrob. Agents Chemother.* **1992,** 36, (12), 2715-2721.

3. Mukherji, E.; Au, J.; Mathes, L. E., Differential antiviral activities and intracellular metabolism of 3'-azido-3'-deoxythymidine and 2', 3'-dideoxyinosine in human cells. *Antimicrob. Agents Chemother.* **1994,** 38, (7), 1573-1579.

4. Tavares, L.; Roneker, C.; Johnston, K.; Lehrman, S. N.; de Noronha, F., 3′-Azido-3′-deoxythymidine in feline leukemia virus-infected cats: a model for therapy and prophylaxis of AIDS. *Cancer Res.* **1987,** 47, (12), 3190-3194.

5. Tavares, L.; Roneker, C.; Postie, L.; de Noronha, F., Testing of nucleoside analogues in cats infected with feline leukemia virus: A model. *Intervirology* **1989,** 30, (Suppl. 1), 26-35.

6. Zeidner, N. S.; Rose, L. M.; Mathiason-DuBard, C. K.; Myles, M. H.; Hill, D. L.; Mullins, J. I.; Hoover, E. A., Zidovudine in combination with alpha interferon and interleukin-2 as prophylactic therapy for FeLV-induced immunodeficiency syndrome (FeLV-FAIDS). *J. Acquir. Immune Defic. Syndr.* **1990,** 3, (8), 787-796.

7. Zeidner, N. S.; Myles, M. H.; Mathiason-DuBard, C. K.; Dreitz, M. J.; Mullins, J. I.; Hoover, E. A., Alpha interferon (2b) in combination with zidovudine for the treatment of presymptomatic feline leukemia virus-induced immunodeficiency syndrome. *Antimicrob. Agents Chemother.* **1990,** 34, (9), 1749-1756.

8. Stuetzer, B.; Brunner, K.; Lutz, H.; Hartmann, K., A trial with 3′-azido-2′, 3′-dideoxythymidine and human interferon-α in cats naturally infected with feline leukaemia virus. *J. Feline Med. Surg.* **2013,** 15, (8), 667-671.

9. Hartmann, K., Efficacy of antiviral chemotherapy for retrovirus-infected cats: What does the current literature tell us? *J. Feline Med. Surg.* **2015,** 17, (11), 925-39.

10. Hartmann, K.; Donath, A.; Kraft, W., AZT in the treatment of feline immunodeficiency virus infection: Part 1. *Feline Pract.* **1995,** 23, 16-21.

11. Hartmann, K.; Donath, A.; Kraft, W., AZT in the treatment of feline immunodeficiency virus infection: Part 2. *Feline Pract.* **1995,** 23, (6), 13-20.

12. Cattori, V.; Weibel, B.; Lutz, H., Inhibition of feline leukemia virus replication by the integrase inhibitor Raltegravir. *Vet. Microbiol.* **2011,** 152, (1-2), 165-8.

13. Boesch, A.; Cattori, V.; Riond, B.; Willi, B.; Meli, M. L.; Rentsch, K. M.; Hosie, M. J.; Hofmann-Lehmann, R.; Lutz, H., Evaluation of the effect of short-term treatment with the integrase inhibitor raltegravir (Isentress) on the course of progressive feline leukemia virus infection. *Vet. Microbiol.* **2015,** 175, (2-4), 167-78.

14. de Mari, K.; Maynard, L.; Sanquer, A.; Lebreux, B.; Eun, H.-M., Therapeutic effects of recombinant feline interferon-w on feline leukemia virus (FeLV)-infected and FeLV/feline immunodeficiency virus (FIV)-coinfected symptomatic cats. *J. Vet. Intern. Med.* **2004,** 18, (4), 477-482.

15. Doménech, A.; Miró, G.; Collado, V. M.; Ballesteros, N.; Sanjosé, L.; Escolar, E.; Martin, S.; Gomez-Lucia, E., Use of recombinant interferon omega in feline retrovirosis: From theory to practice. *Vet. Immunol. Immunopathol.* **2011,** 143, (3), 301-306.

16. Gomez-Lucia, E.; Collado, V. M.; Miro, G.; Martin, S.; Benitez, L.; Domenech, A., Follow-up of viral parameters in FeLV- or FIV-naturally infected cats treated orally with low doses of human interferon alpha. *Viruses* **2019,** 11, (9), 845.

17. Gomez-Lucia, E.; Collado, V. M.; Miró, G.; Martín, S.; Benítez, L.; Doménech, A., Clinical and hematological follow-up of long-term oral therapy with type-I interferon in cats naturally infected with feline leukemia virus or feline immunodeficiency virus. *Animals* **2020,** 10, (9), 1464.

18. Huan, U. E.; Da Silva, P. M. F.; Floriano, A. P., Case studies on RetroMAD1™, an orally administered recombinant chimeric protein to treat naturally infected feline leukemia virus (FeLV) cats. *Arch. Vet. Sci. Med.* **2019,** 2, (4), 41-57.
