# Supplementary material for: Feline leukaemia virus (FeLV) infection in domestic pet cats in Australia and New Zealand: Guidelines for diagnosis, prevention and management
Source: Aust Vet J. 2025 Jul 26;103(10):617–35. doi: 10.1111/avj.13470 (PMC12500364; doi:10.1111/avj.13470)
Supplement: Supplementary file 5 — Table S1. A summary of prices for FeLV testing available to veterinarians in Australia. Prices correct at the time of writing. FIV, feline immunodeficiency virus; NSW, New South Wales; PCR, polymerase chain reaction; PoC, point‐of‐care. [file AVJ-103-617-s006.docx]

**Supplementary Table 1.** A summary of prices for FeLV testing available to veterinarians in Australia. Prices correct at the time of writing. PoC = point-of-care, PCR = polymerase chain reaction, FIV = feline immunodeficiency virus, NSW = New South Wales.

| **Test** | **Laboratory/Manufacturer** | **Cost per test excluding GST (AUD)** |
| --- | --- | --- |
| PoC p27 antigen testing (whole blood, plasma or serum) | | |
| SNAP FIV/FeLV Combo® | IDEXX (Westbrook, ME, USA) | $31.35-43.95 (depending on whether a box of 30, 15 or 5 tests is purchased) |
| Witness® FIV/FeLV | Zoetis Animal Health (Lyon, France) | $27.60 (box of 10) |
| Anigen Rapid® FIV/FeLV | BioNote (Hwaseong-si, Gyeonggido,  Republic of Korea) | $20.80 (box of 10) |
| Proviral DNA PCR testing (whole blood or bone marrow) | | |
| FeLV RealPCR^TM^ | IDEXX  (East Brisbane, Queensland) | $97.50 (or $45.80 following a positive SNAP® p27 antigen result) |
| FeLV PCR | Veterinary Pathology Diagnostic Services (VPDS), University of Sydney (Sydney, NSW) | $81.00 |
| FeLV PCR | Gribbles Veterinary Pathology  (Glenside, South Australia) | $83.75 |
| FeLV PCR | Vetnostics (Macquarie Park, Sydney, NSW) | $113.00 (price also includes FIV PCR testing) |
