# Supplementary material for: Feline leukaemia virus (FeLV) infection in domestic pet cats in Australia and New Zealand: Guidelines for diagnosis, prevention and management
Source: Aust Vet J. 2025 Jul 26;103(10):617–35. doi: 10.1111/avj.13470 (PMC12500364; doi:10.1111/avj.13470)
Supplement: Supplementary file 6 — Table S2. Summary of test results for the different categories of FeLV infection. Neutralising antibody (NAb) testing is currently not available to clinicians in Australia or New Zealand to identify abortive infections. [file AVJ-103-617-s004.docx]

**Supplementary Table 2.** Summary of test results for the different categories of FeLV infection [1-4]. Neutralising antibody (NAb) testing is currently not available to clinicians in Australia or NZ to identify abortive infections.

| Category of FeLV infection | Testing criteria |
| --- | --- |
| Progressive | Persistent p27 antigenaemia and proviral DNA PCR-positivity in blood (i.e., antigen-positive, provirus PCR-positive). |
| Regressive | Initial p27 antigenaemia and proviral DNA PCR-positivity in blood (i.e., antigen-positive, provirus PCR-positive); within 4 months of infection cats become p27 antigen-negative while remaining provirus PCR-positive. |
| Focal | Variable p27 antigenaemia and/or proviral DNA PCR-positivity in blood (i.e., results alternate between negative and positive over time). Sometimes defined as cats that test antigen-positive, provirus PCR-negative. |
| Abortive | Positive neutralising antibody (NAb) titres in the absence of p27 antigenaemia or FeLV provirus (i.e., antigen-negative and provirus PCR-negative, NAb-positive). |

**References**

1. Hofmann-Lehmann, R.; Hartmann, K., Feline leukaemia virus infection: A practical approach to diagnosis. *J. Feline Med. Surg.* **2020,** 22, (9), 831-846.

2. Little, S.; Levy, J.; Hartmann, K.; Hofmann-Lehmann, R.; Hosie, M.; Olah, G.; Denis, K. S., 2020 AAFP feline retrovirus testing and management guidelines. *J. Feline Med. Surg.* **2020,** 22, (1), 5-30.

3. Westman, M.; Norris, J.; Malik, R.; Hofmann-Lehmann, R.; Harvey, A.; McLuckie, A.; Perkins, M.; Schofield, D.; Marcus, A.; McDonald, M.; Ward, M.; Hall, E.; Sheehy, P.; Hosie, M., The diagnosis of feline leukaemia virus (FeLV) infection in owned and group-housed rescue cats in Australia. *Viruses* **2019,** 11, (6), 503.

4. Parr, Y. A.; Beall, M. J.; Levy, J. K.; McDonald, M.; Hamman, N. T.; Willett, B. J.; Hosie, M. J., Measuring the humoral immune response in cats exposed to feline leukaemia virus. *Viruses* **2021,** 13, (3), 428.
